# RORγt phosphorylation protects against T cell-mediated inflammation

**DOI:** 10.1016/j.celrep.2022.110520

**Published:** 2022-03-15

**Authors:** Shengyun Ma, Shefali A. Patel, Yohei Abe, Nicholas Chen, Parth R. Patel, Benjamin S. Cho, Nazia Abbasi, Suling Zeng, Bernd Schnabl, John T. Chang, Wendy Jia Men Huang

**Affiliations:** 1 Department of Cellular and Molecular Medicine, University of California, San Diego, 9500 Gilman Drive, La Jolla, CA 92093, USA; 2 Department of Medicine, University of California, San Diego, 9500 Gilman Drive, La Jolla, CA 92093, USA; 3 Department of Medicine, Veterans Affairs San Diego Healthcare System, San Diego, CA 92161, USA; 4 Lead contact

## Abstract

RAR-related orphan receptor-γ (RORγt) is an essential transcription factor for thymic T cell development, secondary lymphoid tissue organogenesis, and peripheral immune cell differentiation. Serine 182 phosphorylation is a major post-translational modification (PTM) on RORγt. However, the *in vivo* contribution of this PTM in health and disease settings is unclear. We report that this PTM is not involved in thymic T cell development and effector T cell differentiation. Instead, it is a critical regulator of inflammation downstream of IL-1β signaling and extracellular signal regulated kinases (ERKs) activation. ERKs phosphorylation of serine 182 on RORgt serves to simultaneously restrict Th17 hyperactivation and promote anti-inflammatory cytokine IL-10 production in RORγt^+^ Treg cells. Phospho-null RORγt^S182A^ knockin mice experience exacerbated inflammation in models of colitis and experimental autoimmune encephalomyelitis (EAE). In summary, the IL-1β-ERK-RORγt^S182^ circuit protects against T cell-mediated inflammation and provides potential therapeutic targets to combat autoimmune diseases.

## In brief

A balanced mucosal T cell population is essential for tissue homeostasis and wound healing post-injury and infection. In this study, Ma et al. report a surprising role for the phosphorylated transcription factor RORγt as a cell-intrinsic regulator for maintaining mucosal T cell heterogeneity and promoting inflammation resolution.

## Graphical Abstract

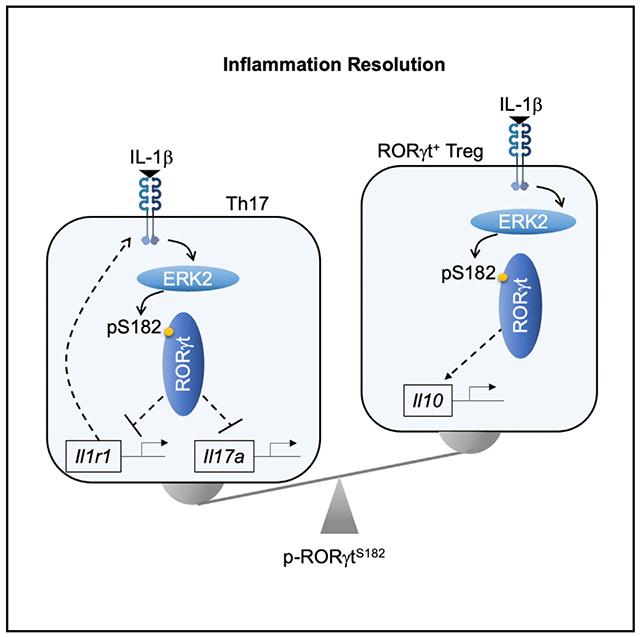

## INTRODUCTION

The evolutionarily conserved *RORC* locus encodes two isoforms of transcription factor called RAR-related orphan receptor-γ (RORγ). The long isoform (RORγ) is broadly expressed, but the shorter isoform (RORγt) is found in cells of the immune system (Jetten and Joo, 2006). Humans with *RORC* loss-of-function mutations have impaired antibacterial and antifungal immunity (Okada et al., 2015). Genetic deletion and pharmacologic studies using mouse models demonstrate the essential roles of RORγt in secondary lymphoid tissue (LT) organogenesis (Cherrier et al., 2012) and the differentiation of type 3 innate lymphoid cells (ILC3s) and effector T lymphocytes, including T helper 17 (Th17), Th1-Th17, RORγt^+^ Treg (T regulatory) or Tγδ17 cells (Ivanov et al., 2006; Kim et al., 2017; Scoville et al., 2016). Genetic deletion or pharmacologic inhibition of RORγt protects against autoimmune diseases (Ivanov et al., 2006; Kim et al., 2017; Sun et al., 2019; Yang et al., 2015), while RORγt overexpressing transgenic mice develop greater susceptibility (Martinez et al., 2014). As many of the RORγt-dependent immune populations are ablated in the RORγt knockout mice, the current knowledge of RORγt functions in mature immune cells is based on findings from *in vitro* cell culture studies. Therefore, the exact *in vivo* functions of RORγt and the mechanisms by which it controls cell-type-specific gene programs in the various mature effector immune subsets remain to be elucidated.

The RORγt protein consists of a DNA-binding domain (DBD), a hinge region, and a ligand-binding domain (LBD). While the importance of DBD and LBD post-translational modifications (PTMs) on RORγt expression and function are well appreciated (reviewed in Rutz et al., 2016), the contribution of the hinge region and its PTMs remains largely unexplored. A recent report suggests that the hinge region of RORγt is involved in the organogenesis of Peyer’s patches and Th17 differentiation, but is dispensable for thymic T cell development and lymph node biogenesis (He et al., 2017a). Here, we demonstrate that the phosphorylation of the evolutionarily conserved serine 182 on the hinge region is the most abundant RORγt PTM and is mediated by extracellular signal regulated kinases (ERKs) downstream of interleukin-1 β (IL-1β) signaling.

Elevated IL-1β is associated with increased disease severity in patients with inflammatory bowel diseases (Coccia et al., 2012; Ligumsky et al., 1990). In dextran sodium sulfate (DSS) and infection-induced mouse models of colitis, dysregulated IL-1β, and/or its receptor signaling also promote exacerbated tissue inflammation (Maeda et al., 2005; Muller et al., 2009; Neudecker et al., 2017; Ng et al., 2010; Saitoh et al., 2008; Seo et al., 2015). IL-1β signals via the IL-1R1 and IL-1R3 complexes (referred to as IL-1R) and activate the mitogen-activated protein kinase (MAPK) pathway. IL-1β and MAPKs, including ERK, Jun N-terminal kinase (JNK), and p38, play important roles in T cell development, activation, differentiation, and effector functions (Fischer et al., 2005; Jain et al., 2018; Van Den Eeckhout et al., 2020). ERKs negatively regulates Th17 generation and effector function in autoimmunity diseases (Cui et al., 2009; Zhang et al., 2015). Pharmacologic inhibition of ERK signaling enhances Th17 differentiation (Tan and Lam, 2010; Wu et al., 2021) and exacerbates disease in a model of T cell transfer colitis (Tan and Lam, 2010). When challenged in the experimental autoimmune encephalomyelitis (EAE) model, mice deficient in ERK1 have an earlier disease onset and an increased disease severity (Agrawal et al., 2006). ERKs have also been reported to regulate IL-10 expression in Treg cells (Liu et al., 2013). However, the downstream phosphorylation targets of ERKs in diverse T cell populations remain poorly characterized.

Here, we report that IL-1β signaling promotes the ERK phosphorylation of RORγt^S182^ to simultaneously restrict pathogenic Th17 effector functions, potentiate RORγt^+^ Treg programs, and resolve tissue inflammation. Genetic and pharmacologic inhibition of the IL-1β-ERK-RORγt^S182^ circuit exacerbates tissue inflammation in the intestine. Single-cell transcriptomic (scRNA sequencing [RNA-seq]) analyses further reveal RORγt^S182^-dependent metabolic and stress response programs and their critical role in maintaining intestinal T cell heterogeneity. Importantly, this circuit is dispensable for normal thymic T cell development and peripheral effector cell differentiation and provides potential targets for ameliorating T cell-mediated autoimmunity.

## RESULTS

### RORβt^S182^ is dispensable for normal thymic T cell development *in vivo*

**Figure 1.**
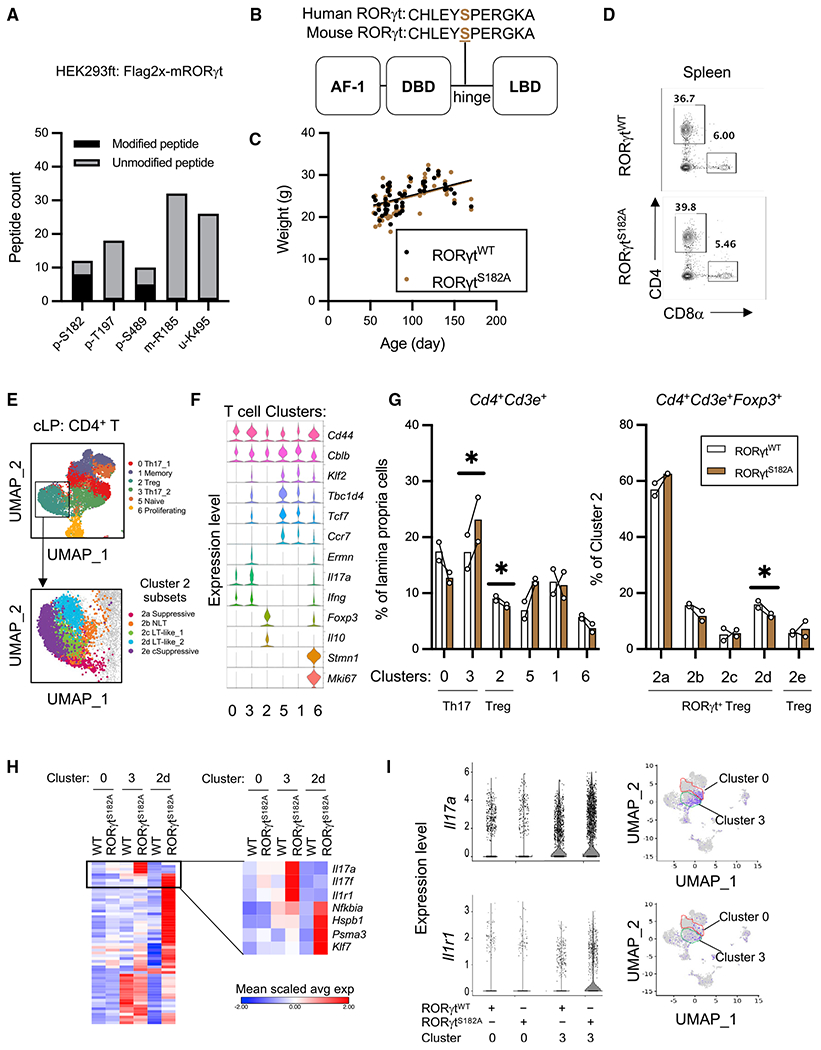
Normal T cell development and differentiation in RORγt^S182A^ mice (A) Proportion of modified and unmodified peptides identified by tandem mass spectrometry (MS/MS) mapping to murine RORγt from whole-cell lysates (WCL) of HEK293 cells transfected with a 2xFLAG-mRORγt expression construct for 48 h. Phosphorylation (p), methylation (m), and ubiquitination (u). (B) Model diagram of RORγt protein domains. Black vertical line indicates the position of the evolutionarily conserved serine 182. AF-1, activation function domain 1; DBD, DNA-binding domain; LBD, ligand-binding domain. (C) Weights of 2- to 4-month-old wild-type (RORγt^WT^, n = 49) and RORγt^S182A^ (n = 57) adult mice obtained from heterozygous crosses. Each dot represents the result from 1 mouse. (D) Representative flow cytometry analysis of cell surface CD4 and CD8α on splenocytes from RORγt^WT^ and RORγt^S182A^ cohoused littermates. This experiment was repeated 3 times on independent biological samples with similar results. (E) UMAP plots depicting the transcriptomes of immune cell clusters (top) and cluster 2 subsets (bottom) obtained from colonic lamina propria (cLP) CD4^+^ T cells (10X Genomics droplet-based 3′ scRNA-seq). (F) Violin plots of selected gene expressions in cells from the indicated clusters. (G) Proportions of colonic *Cd4*^+^*Cd3e*^+^ (left) and *Cd4*^+^*Cd3e*^+^*Foxp3*^+^ (right, cluster 2) T cells in each cluster from 2 pairs of RORγt^WT^ and RORγt^S182A^ littermates. Each bar represents the sample mean. * p < 0.05 (ratio paired t test). (H) Heatmap of mean scaled average expression of selected genes from the indicated clusters. (I) Violin plots (left) and UMAP (right) showing expression of *Il17a* and *Il1r1* in colonic Th17 cell subsets (clusters 0 and 3) in steady-state RORγt^WT^ and RORγt^S182A^ mice.

The characterization of RORγt PTMs by immunoprecipitation and tandem mass spectrometry (MS/MS) revealed phosphorylation at the evolutionarily conserved serine 182 as the most prominent PTM on RORγt (Figures 1A, 1B, and S1A), which is consistent with previous reports (He et al., 2017b; Huttlin et al., 2010). To assess the *in vivo* function of this PTM, we generated a phospho-null knockin C57BL/6 mouse line (RORγt^S182A^) by replacing the serine codon on *Rorc* with that of alanine using CRISPR-Cas9 technology (Figure S1B). Heterozygous crosses yielded control (RORγt^WT^) and homozygous RORγt^S182A^ littermates in a Mendelian ratio with similar growth rates under cohoused conditions (Figure 1C). Unlike the *RORγt^−/−^* mice that were reported to have impaired thymic T cell development due to the reduced expression of an anti-apoptotic factor B cell lymphoma-extra large (Bcl-xL) in the CD4^+^CD8^+^ (double positive [DP]) cells (Guo et al., 2016; Sun et al., 2000), DP thymocytes in the RORγt^S182A^ mice expressed normal levels of RORγt and Bcl-xL proteins (Figures S1C–S1E). Mature RORγt^S182A^ single-positive CD4^+^ T helper and CD8^+^ cytotoxic T cells trafficking to the spleen also appeared normal (Figure 1D). These *in vivo* results suggest that RORγt^S182A^ can replace wild-type (WT) RORγt proteins to support normal thymic T cell development under steady state.

### RORγt^S182^-dependent Th17 and RORγt^+^ Treg programs in the colon

In the steady-state intestinal lamina propria, RORγt is known to be important for the differentiation and functions of multiple immune cell subsets, including Th17, RORγt^+^ Tregs or Tγδ17, and ILC3s. Flow cytometry revealed similar proportions of RORγt-expressing Th17, RORγt^+^ Tregs or Tγδ17, and ILC3s in the small intestine and colon of steady-state control and RORγt^S182A^ mice (Figures S2A and S2B). In total lamina propria cell lysates, we found a similar expression of RORγt-regulated genes encoding IL-17A and IL-10 cytokines at the RNA and protein levels (Figures S3A and S3B). Recent studies implied that intestinal T cells can be further divided into diverse subsets that cannot be illuminated by transcription factor staining alone (Huang et al., 2021; Schnell et al., 2021). To better assess the contribution of RORγt^S182^ to the T cell heterogeneity *in vivo*, we performed scRNA-seq analysis on 10,000–13,000 CD4^+^ T cells from colonic lamina propria (cLP) in 2 pairs of RORγt™^WT^ and RORγt^S182A^ cohoused littermates (Figure S3C). Uniform manifold approximation and projection (UMAP) of 11,725 cells with *Cd4* > 0.4 and ~1,100 genes per cell revealed 12 cell clusters. In-depth analysis was performed on 6 main clusters (consisting of 8,175 cells, 69.7% total) with high co-expression of *Cd4* and *Cd3e*, including 2 subsets of Th17 (cluster 0 and 3, *Il17a^high^*), memory T cells (cluster 1, *Cd44*^high^), Treg cells (cluster 2, *Foxp3*^high^*Il10*^high^), naive T cells (cluster 5, *Cd44*^low^), and proliferating T cells (cluster 6, *Ki67*^high^) (Figures 1E, 1F, and S3D). Of the 2 closely related Th17 subsets, cluster 0 cells expressed higher levels of *Cxcl1*, *Ccl8*, *Cxcl16*, and *Cxcl13*, encoding chemotactic factors for neutrophils, monocytes, macrophages, and B cells, respectively. In contrast, cluster 3 cells had higher levels of *Ccl20*, *Il23r*, and *Il22*, encoding chemotactic factors for lymphocytes, receptors for IL-23A, and regulators of intestinal epithelial cell regeneration and maturation, respectively (Figure S3E). In RORγt^WT^ steady-state colons, these 2 Th17 subsets were present in a balanced 1:1 ratio (Figure 1G). In the RORγt^S182A^ colon, however, this balance was disrupted with the dominance of cluster 3 Th17 cells.

In addition to the altered Th17 populations, the RORγt^S182A^ steady-state colon also harbored fewer *Foxp3*^high^ regulatory T (Treg) cells (cluster 2), including 2 suppressive Tregs (2a and 2e, *Gzmb*^high^ *Ccr1*^high^), two LT-like Tregs (2c and 2d, *S1pr1*^high^), and a non-LT Treg (2b, *Gata3*^high^ *Pdcd1*^high^) population (Figure 1E). Four of five Treg populations expressed modest to high levels of *Rorc* (subsets 2a–2d) likely represent various RORγt^+^ Treg populations (Figure S3F). In the RORγt^S182A^ colon, the proportion of the cluster 2d cells (*S1pr1*^high^*Itgal*^high^*Tcf7*^high^ LT like) was greatly reduced (Figure 1G). No significant differences in the proportions of other T cell populations were found. These results suggest that RORγt^S182^ is essential for maintaining proper colonic Th17 and RORγt^+^ Treg subset heterogeneity under steady state.

### RORγt^S182A^ mutant mice develop exacerbated colitis when challenged with DSS

**Figure 2.**
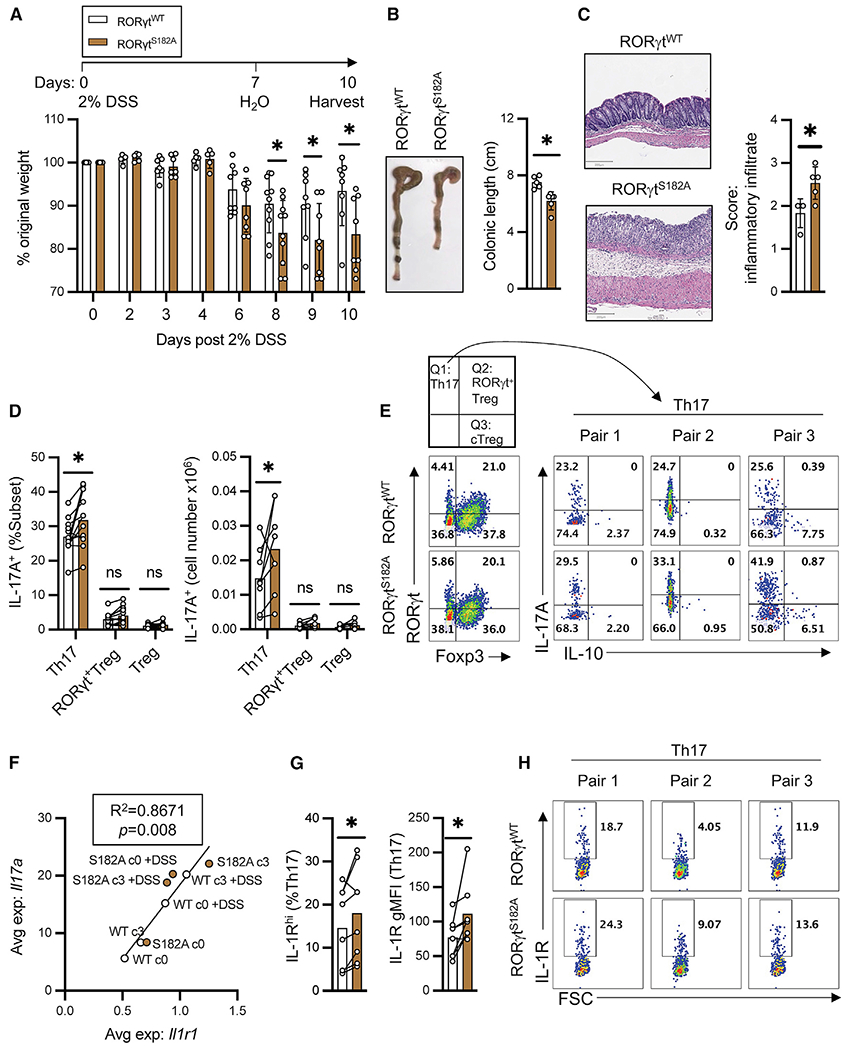
RORγt^S182A^ mice have exacerbated diseases in DSS-induced colitis (A) Weight changes of RORγt^WT^ and RORγt^S182A^ cohoused littermates challenged with 2% DSS in drinking water for 7 days and monitored for another 3 days. Each bar represents the sample mean. Each dot represents the result from 1 mouse. * p < 0.05 (multiple t test). (B) Left: Representative bright-field images of colons from (A). Right: Summarized colonic lengths of DSS-treated RORγt^WT^ (n = 5) and RORγt^S182A^ (n = 6) mice harvested on day 10. * p < 0.05 (t test). (C) Left: Representative colonic sections from (B). Right: Summarized score of colonic inflammatory infiltrates in DSS-treated RORγt^WT^ (n = 4) and RORγt^S182A^ (n = 5) mice. Each bar represents the sample mean. * p < 0.05 (t test). (D) Proportion and absolute number of IL-17A^+^ producing Th17 (T cell receptor [TCR]β^+^RORγt^+^Foxp3^−^), RORγt^+^ Treg (TCRβ^+^RORγt^+^Foxp3^+^), and conventional Tregs (TCRβ^+^RORγt^−^Foxp3^+^) in colons from DSS-challenged RORγt^WT^ and RORγt^S182A^ mice. Each dot represents the result from 1 mouse. Each bar represents the sample mean. *p < 0.05; n.s., not significant (paired t test). (E) Representative flow cytometry analysis of colonic CD4^+^ T subsets and IL-17A and IL-10 production capacities in Th17 cells from DSS-treated mice. (F) Linear regression analysis of *Il17a* and *Il1r1* average expressions in colonic Th17 subsets from the indicated conditions as determined by scRNA-seq. (G) IL-1R^high^ proportion and IL-1R geometric mean fluorescence (gMFI) in colonic RORγt^WT^ and RORγt^S182A^ Th17 cells from DSS-challenged mice. Each dot represents the result from 1 mouse. Each bar represents the sample mean. * p < 0.05 (paired t test). (H) Representative flow cytometry analysis of IL-1R in colonic Th17 cells from DSS-challenged mice harvested on day 10. Forward scatter (FSC).

Despite the altered steady-state T cell heterogeneity and transcriptomes in the colon, we did not observe any changes to the IL-17A^+^ proportion of total Th17 cells in the steady-state colon by flow cytometry (Figures S3A and S3B). RORγt^S182A^ mice displayed growth rates similar to those of control littermates and did not develop spontaneous colitis (Figure 1B). Nonetheless, we were intrigued about the augmented levels of *Il17a* and *Il1r1* transcripts found in cluster 3 RORγt^S182A^ colonic Th17 cells (Figures 1H and 1I), as the elevated IL-1β level was associated with increased disease severity in patients with inflammatory bowel diseases (Coccia et al., 2012; Ligumsky et al., 1990). In DSS- and infection-induced mouse models of colitis, dysregulated IL-1β and/or its receptor signaling also promoted exacerbated tissue inflammation (Maeda et al., 2005; Muller et al., 2009; Neudecker et al., 2017; Ng et al., 2010; Saitoh et al., 2008; Seo et al., 2015). Therefore, we hypothesized that elevated *Il17a* and *Il1r1* transcripts in colonic Th17 cells may position RORγt^S182A^ mice to develop exacerbated tissue inflammation in response to elevated IL-1β signaling during DSS-induced colitis. Indeed, DSS-challenged RORγt^S182A^ mice experienced a significant delay in weight recovery as compared to their cohoused RORγt^WT^ littermates (Figure 2A). The colons harvested from DSS-challenged RORγt^S182A^ mice were shorter than those from their DSS-challenged RORγt^WT^ littermates (Figure 2B). Histology confirmed an increase in infiltrated immune cells present in the colons from DSS-challenged RORγt^S182A^ mice (Figure 2C). These results suggest that RORγt^S182^ is implicated in protecting intestine mucosa from exacerbated inflammation post-DSS-induced epithelial injury.

Total cLP lysates from DSS-challenged RORγt^S182A^ mice showed similar levels of *Il17a* and a modest reduction in *Il10* (Figure S4A). To elucidate RORγt^S182^-dependent gene program-specific T cell subsets in the inflamed tissues, we performed scRNA-seq analysis on CD4^+^ T cells from cLP in 2 pairs of RORγt^WT^ and RORγt^S182A^ cohoused littermates 10 days post-DSS challenge. Differential gene expression analysis revealed a surprising role for RORγt^S182^ in regulating the colonic Th17 transcriptomes under both steady-state and DSS-challenged conditions. Group II genes, including *Il17a*, were previously reported to be downregulated in *ROR*γ*t*^−/−^ cultured Th17 cells (Ciofani et al., 2012) and were found to be upregulated in colonic RORγt^S182A^ Th17 cells from steady-state and DSS-challenged mice (Figure S4B). Flow cytometry analysis confirmed that there were a greater proportion and an absolute number of IL-17A-producing colonic Th17 cells in DSS-challenged RORγt^S182A^ mice (Figures 2D and 2E). The elevated *Il17a* level was strongly associated with the higher expression of *Il1r1* transcript (Figure 2F) and cell surface IL-1R (Figures 2G and 2H). Importantly, these changes were not due to altered RORγt protein levels in the mutant CD4^+^ T cells (Figures S4C and S4D).

**Figure 3.**
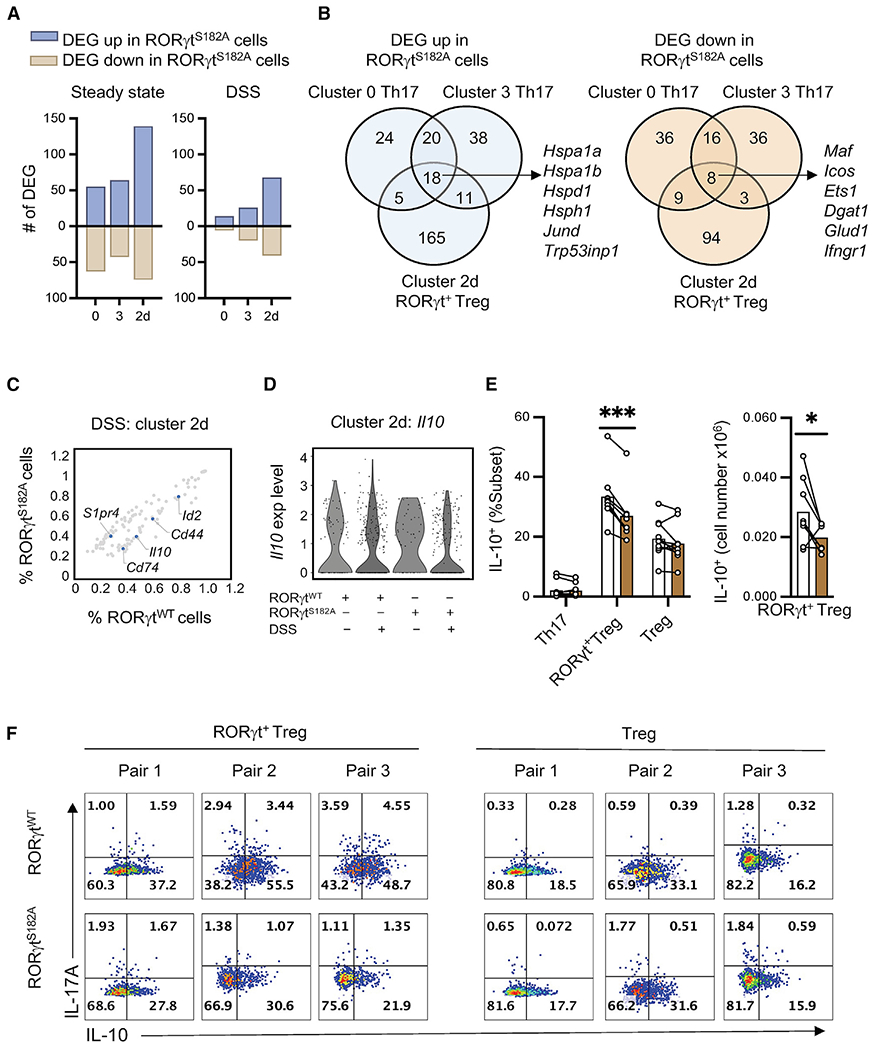
Common and distinct RORγt^S182^-dependent gene programs in colonic Th17 and RORγt^+^ Treg cells (A) Number of RORγt^S182^-dependent genes (DEG, p < 0.05) in colonic Th17 (cluster 0 and 3) and RORγt^+^ Treg cells (cluster 2d) from steady-state or DSS-challenged mice. (B) Venn diagram showing overlap and subset-specific RORγt^S182^-regulated genes in Th17 and RORγt^+^ Treg cells from (A). (C) Percentage of cells expressing RORγt^S182^-dependent genes (gray dots, p < 0.05) in colonic RORγt^+^ Treg cells (subset 2d) from DSS-challenged RORγt^WT^ and RORγt^S182A^ mice. Select genes were labeled and highlighted in blue. (D) Violin plot of *Il10* expression in RORγt^+^ Treg cells (subset 2d) from control or DSS-challenged RORγt^WT^ and RORγt^S182A^ mice. (E) Proportion and cell number of IL-10-producing colonic Th17, RORγt^+^ Treg, and conventional Treg cells from DSS-challenged RORγt^WT^ and RORγt^S182A^ mice. Each dot represents the result from 1 mouse. Each bar represents the sample mean. *p < 0.05; ***p < 0.001; n.s., not significant (paired multiple t test). (F) Representative flow cytometry analysis of IL-17A and IL-10 in colonic RORγt^+^ Treg and conventional Treg cells from DSS-challenged mice harvested on day 10.

scRNA-seq differential gene expression analysis of all 3 RORγt^S182^-dependent T cell subsets (clusters 0, 3, and 2d, as defined in Figure 1F) further uncovered common and subset-specific transcripts regulated by RORγt^S182^ under steady-state and/or DSS-challenged conditions. Shared among all 3 subsets were 18 (6.4%) genes upregulated and 8 (4%) genes downregulated in RORγt^S182A^ cells, many encoding molecules involved in stress response (*Hspa1a* and *Hspa1b*) and metabolism (*Dgat1* and *Glud1*) (Figures 3A and 3B). Importantly, many of these *in vivo*-identified RORγt targets had not been reported in prior cell culture-based studies.

In RORγt^+^ Tregs (subset 2d) from the DSS-challenged colon, *Il10* transcripts and protein abundance were downregulated in cells expressing RORγt^S182A^ (Figures 3C–3F). In contrast, no change in IL-10 production potential was observed in conventional Tregs. The reduction in *Il10* transcript abundance in mutant RORγt^+^ Tregs was accompanied by the loss of *Maf*, *Cd44*, and *Id2* transcripts (Figure 3B) encoding molecules previously implicated in Treg activation and IL-10 production (Bollyky et al., 2009; Hwang et al., 2018; Wheaton et al., 2017), as well as an increased expression of *S1pr4*, which is involved in cell migration (Olesch et al., 2017). Collectively, these results suggest that serine 182 on RORγt regulates common and distinct gene programs in colonic Th17 and RORγt^+^ Treg subsets.

### T cell-intrinsic role of RORγt^S182^ in a model of transfer colitis

**Figure 4.**
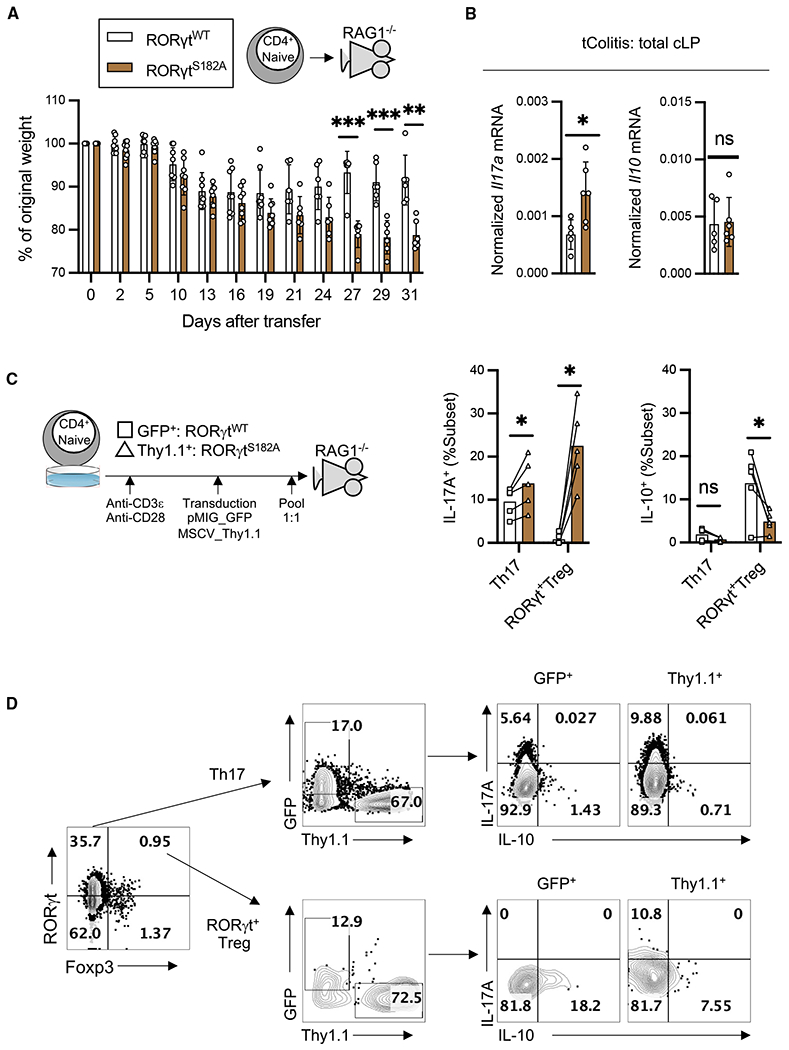
Transfer of RORγt^S182A^ CD4^+^ T cells promote exacerbated wasting in Rag1^−/−^ mice (A) Weight changes of *Rag1*^−/−^ mice receiving RORγt^WT^ (n = 6) or RORγt^S182A^ (n = 6) naive CD4^+^ T cells. Each dot represents the result from 1 mouse. Each bar represents the sample mean. **p < 0.01 and ***p < 0.001 (multiple t test). (B) RNA expression level of *Il17a* and *Il10* in total cLP cell lysate from *Rag1*^−/−^ mice in (A). Each dot represents the result from 1 mouse. Each bar represents the sample mean. *p < 0.05; n.s., not significant (t test). (C) Right: Activated RORγt^WT^ (transduced with GFP expressing pMIG construct) or RORγt^S182A^ (transduced with Thy1.1 expressing MSCV construct) CD4^+^ T cells were mixed in a 1:1 ratio and injected into *Rag1*^−/−^ mice. Left: Summarized proportion of IL-17A^+^ and IL-10^+^ colonic Th17 and RORγt^+^ Treg cells from *Rag*^−/−^ recipients 33 days post-transfer (n = 5). Each line represents results from same *Rag1*^−/−^ recipient. Each bar represents the mean from 5 *Rag1*^−/−^ recipients. *p < 0.05; n.s., not significant (multiple t test). (D) Representative flow cytometry analysis of IL-17A and IL-10 in colonic Th17 and RORγt^+^ Treg cells from (C).

To test whether the protective role of RORγt^S182^ during inflammatory challenge is due to its expression in CD4^+^ T cells, RORγt^WT^ or RORγt^S182A^ CD4^+^-naive T cells were introduced into recombination activating gene (*RAG*)*1*^−/−^ recipients in a model of T cell-mediated colitis. For the first 2 weeks post-transfer, recipients of RORγt^WT^ or RORγt^S182A^ cells experienced similar levels of weight loss. By day 19, conditions in the recipients of RORγt^WT^ cells were stabilized, but recipients of RORγt^S182A^ cells continued to lose weight (Figure 4A). Total cLP lysates from RORγt^S182A^ T cell recipients showed reduced expression of *Il17a*, but similar levels of *Il10* (Figure 4B). cLP and spleens in the recipients of RORγt^S182A^ T cells harbored a greater number of IL-17A-producing Th17 cells (Figures S5A and S5B). Thus, RORγt^S182^ protects against CD4^+^ T cell mediated colitis .

To assess whether RORγt^S182^ regulation of T cell cytokine production is cell intrinsic, we co-transferred RORγt^WT^ and RORγt^S182A^ CD4^+^ T cells marked by GFP and Thy1.1, respectively, into *Rag1*^−/−^ recipients. One month post-transfer, colonic RORγt^S182A^T cells had higher IL-17Aand lower IL-10 production potentials compared to RORγt^WT^ cells in the same tissue (Figures 4C and 4D). Taken together, these *in vivo* results demonstrate a T cell-intrinsic role of S182 on RORγt in protecting against inflammatory conditions in the colonic mucosa.

### Exacerbated central nervous system (CNS) inflammation in RORγt^S182A^ mice challenged in the EAE model

**Figure 5.**
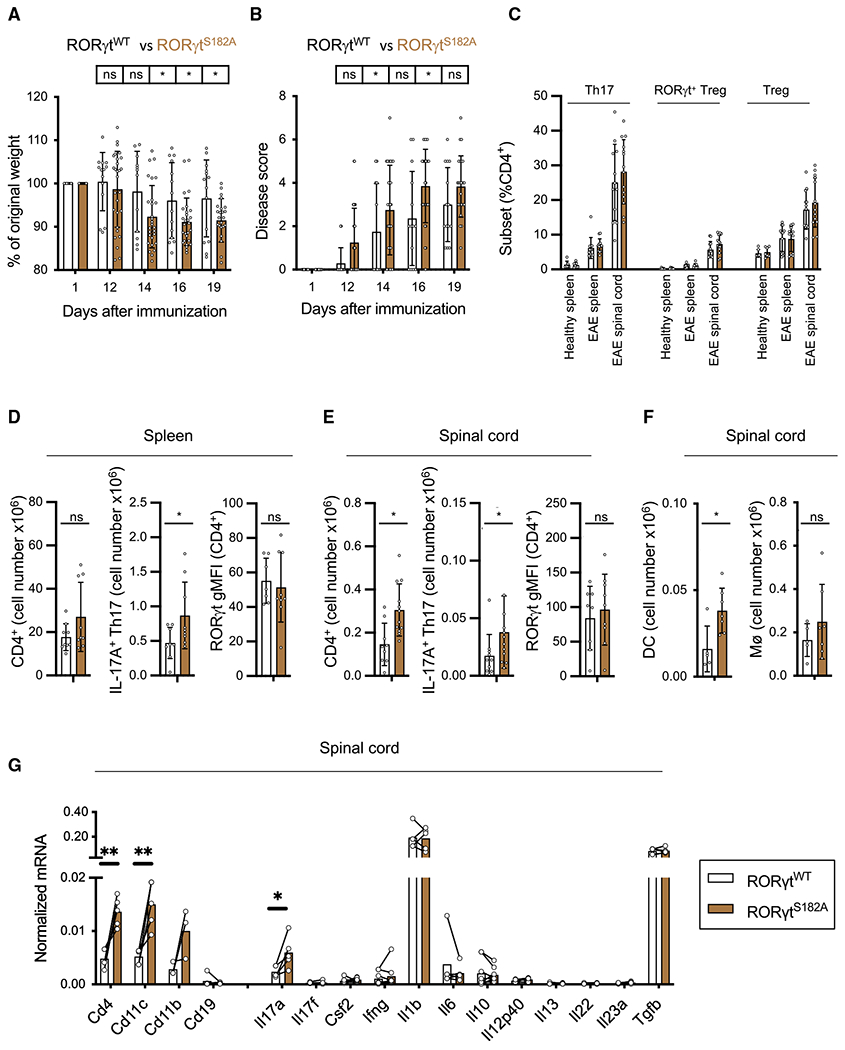
RORγt^S182A^ mice challenged in the EAE model experienced more severe disease (A) Weight change of MOG-immunized RORγt^WT^ (n = 14) and RORγt^S182A^ (n = 23) mice. Each dot represents the result from 1 mouse. Each bar represents the sample mean. * p < 0.05; n.s., not significant (multiple t test). (B) Disease score of mice from (A). Each dot represents the result from 1 mouse. Each bar represents the sample mean. *p < 0.05; n.s., not significant (multiple t test). (C) Proportion of Th17, RORγt^+^ Treg, and conventional Treg cells in healthy spleens as well as spleens and spinal cords from MOG-immunized RORγt^WT^ and RORγt^S182A^ mice. Each dot represents the result from 1 mouse. Each bar represents the sample mean. (D) Cell numbers of CD4^+^ and IL-17A^+^ Th17 cells, and gMFI of RORγt in the spleens of MOG-immunized mice from (A) harvested at the peak of disease (day 19). Each dot represents the result from 1 mouse. Each bar represents the sample mean. *p < 0.05; n.s., not significant (t test). (E) Cell numbers of CD4^+^ and IL-17A^+^Th17 cells and gMFI of RORγt in the spinal cord MOG-immunized mice from (A) harvested at the peak of disease (day 19). Each dot represents the result from 1 mouse. Each bar represents the sample mean. *p < 0.05; n.s., not significant (t test). (F) Cell numbers of macrophages (MØ, CD11b^+^F4/80^+^) and dendritic cells (DC, CD3ε^−^CD11c^+^) in the spinal cord of MOG-immunized mice harvested at the peak of disease (day 19). Each dot represents the result from 1 mouse. Each bar represents the sample mean. *p < 0.05; n.s., not significant (t test). (G) Normalized mRNA expression of select genes in the immune infiltrates of the spinal cords at the peak of EAE disease from MOG-immunized RORγt^WT^ and RORγt^S182A^ mice as detected by qRT-PCR. Each dot represents the result from 1 mouse. Each bar represents the sample mean. *p < 0.05, **p < 0.01 (paired multiple t test).

In addition to the pathogenic role during intestine inflammation, Th17 cells are one of the major contributors to CNS inflammation (Cua et al., 2003; Ivanov et al., 2006; Solt et al., 2011; Xiao et al., 2014). Therefore, we next asked whether RORγt^S182^ and its phosphorylation were also involved in EAE pathogenesis. Myelin/oligodendrocyte glycoprotein (MOG)-immunized RORγt^S182A^ mice experienced more weight loss and developed more mobility issues than MOG-immunized control littermates (Figures 5A and 5B). At the cellular level, however, we observed a more consistent response to MOG immunization. In both the spleens and spinal cords of RORγt^WT^ and RORγt^S182A^ mice, MOG immunization resulted in increased Th17, RORγt+ Treg, and conventional Treg proportions (Figure 5C). MOG-challenged RORγt^S182A^ mice also harbored higher number of IL-17A-producing Th17 cells in the spleens and spinal cords (Figures 5D and 5E), as well as an increase in total CD4^+^ T cells and CD11c^+^ dendritic cells in the spinal cords (Figures 5E and 5F). These differences in the spinal cords were also observed at the RNA level (Figure 5G). Results from these EAE experiments suggest that the protective role of RORγt^S182^ can be extended to T cell-mediated inflammation in the CNS.

### RORγt^S182^ modulates IL-1β-dependent cytokine productions in cultured Th17 cells

**Figure 6.**
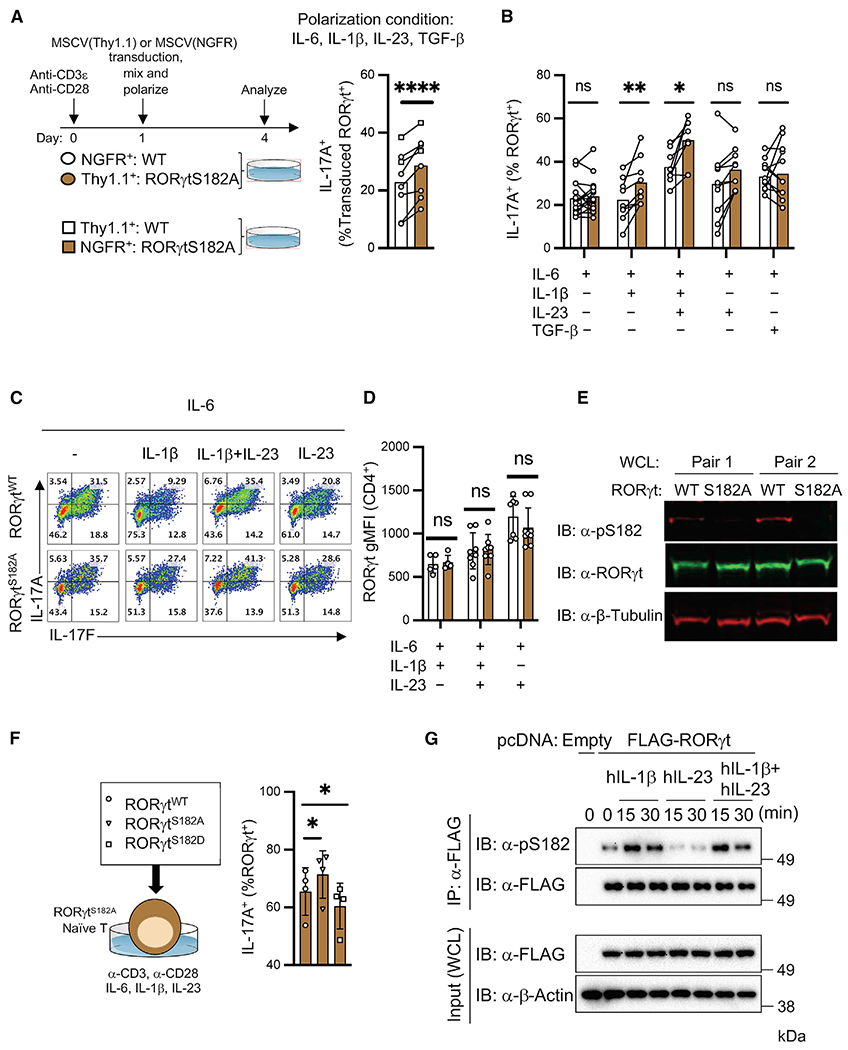
Cultured RORγt^S182A^ Th17 cells harbor augmented cytokine production potential in response to IL-1β signaling (A) Left: Workflow of the co-culture experiment using the indicated transduced vectors and marked RORγt^WT^ and RORγt^S182A^ CD4^+^ T cells polarized in the presence of IL-6 (20 ng/mL), IL-1β (20 ng/mL), IL-23 (25 ng/mL), and TGF-β (0.3 ng/mL). Right: Summarized proportion of IL-17A^+^ in cultured cells 3 days postpolarization. Each line represents results from 1 experimental well. ****p < 0.0001 (paired t test). (B) Summarized proportion of IL-17A^+^ in Th17 cells cultured in the indicated conditions. Each dot represents the result from 1 mouse. Each bar represents the sample mean. *p < 0.05, **p < 0.01; n.s., not significant (paired multiple t test). (C) Representative flow cytometry analysis of IL-17A and IL-17F expression in cultured Th17 cells from (B). (D) RORγt gMFI in cultured Th17 cells from (B). Each dot represents the result from an independent experiment. Each bar represents the sample mean. n.s., not significant. (E) Immunoblot (IB) analysis of total RORγt or RORγt phosphorylated at S182 (pS182) in cultured Th17 WCL from 2 pairs of RORγt^WT^ and RORγt^S182A^ littermates. (F) Proportion of IL-17A^+^ among cultured RORγt^S182A^ Th17 cells transduced with retroviruses carrying RORγt^WT^ (circle) (Huh et al., 2011), RORγt^S182A^ (triangle), or RORγt^S182D^ (square) expression constructs. Each dot represents the result from an independent experiment. Each bar represents the sample mean. *p < 0.05 (t test). (G) HEK293 cells transfected with empty or 2xFLAG-RORγt expression constructs for 16 h were stimulated with the indicated cytokines. RORγt was captured by anti-FLAG beads and pS182 status was detected by immunoblot.

To dissect the molecular mechanism(s) underlying RORγt^S182^ regulation of Th17 cell functions, we used a culture system in which naive RORγt^WT^ and RORγt^S182A^ CD4^+^ T cells marked by nerve growth factor receptor (NGFR) and/or Thy1.1 were cocultured and polarized in the presence of cytokines found in inflamed tissues, including IL-6, IL-1 β, IL-23, and transforming growth factor-β (TGF-β). Consistent with *in vivo* observations, cultured cells with distinct genotypes continued to maintain differential IL-17A expression (Figure 6A), suggesting that serine 182 on RORγt is a cell-intrinsic regulator of Th17 cytokine production.

To delineate which stimuli was upstream of the RORγt^S182^-dependent pathway, we tested different combination of the polarizing condition and found that IL-6 in combination with IL-1β, in the presence or absence of IL-23, was necessary for engaging the RORγt^S182^ axis in fine-tuning IL-17A production in cultured Th17 cells (Figures 6B and 6C). IL-6 in combination with IL-23 or TGF-β did not trigger similar responses. Whole-cell lysates (WCLs) also confirmed that S182 on RORγt was phosphorylated in these cultured T cells and that the overall RORγt protein abundance in control and mutant cells was comparable (Figures 6D and 6E). As expected, expression of the phosphomimic mutant (RORγt^S182D^), but not WT or phospho-null proteins, restricted IL-17A production capacity in cultured RORγt^S182A^ Th17 cells (Figure 6F).

Furthermore, biochemical assays using human HEK293 cells transiently transfected with FLAG-tagged RORγt expression construct confirmed that IL-1β, but not IL-23, stimulation alone was sufficient to promote S182 phosphorylation on RORγt (Figure 6G). These results suggest that IL-1β-driven S182 phosphorylation on RORγt is likely evolutionarily conserved.

### ERK2 phosphorylation of RORγt protects against exacerbated DSS-induced colitis

**Figure 7.**
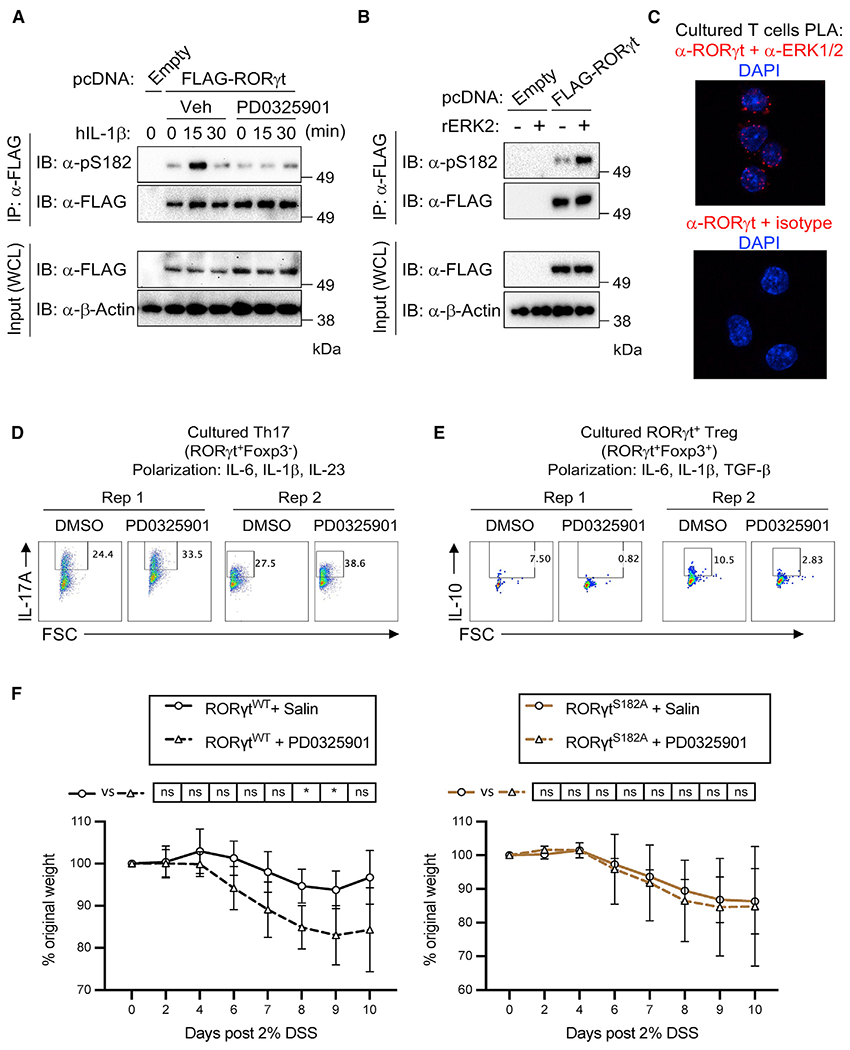
ERK2 phosphorylation of RORγt protects against exacerbated DSS-induced colitis (A) HEK293 cells transfected with empty or 2xFLAG-RORγt expression constructs for 16 h were treated with dimethylsulfoxide (DMSO, Veh) or ERK inhibitor (PD0325901, 5μM) and stimulated with the indicated cytokines. RORγt was captured by anti-FLAG beads and pS182 status was detected by immunoblot. (B) HEK293 cells transfected with empty or 2xFLAG-RORγt expression constructs for 16 h. RORγt was captured by anti-FLAG beads and incubated with recombinant ERK2 *in vitro*. Phospho-S182 status was detected by immunoblot. This experiment was repeated twice with similar results. (C) Representative images from proximity ligation assay (PLA) indicating interactions between RORγt and ERK1/2 in wild-type cultured Th17 cells. (D) Representative flow cytometry analysis of IL-17A expression in wild-type Th17 cells polarized under IL-6 (20 ng/mL), IL-1β (20 ng/mL), and IL-23 (25 ng/mL) in the presence or absence of ERK inhibitor (PD0325901, 5μM) at 48 h. Cells were harvested and analyzed at 72 h. (E) Representative flow cytometry analysis of IL-10 expression in wild-type RORγt^+^ Treg cells polarized under IL-6 (20 ng/mL), IL-1β (20 ng/mL), and TGF-β (5 ng/mL) in the presence or absence of ERK inhibitor (PD0325901, 5μM) at 48 h. Cells were harvested and analyzed at 72 h. (F) Weight changes in RORγt^WT^ (left) and RORγt^S182A^ (right) cohoused littermates challenged with 2% DSS in drinking water for 7 days, followed by regular water for 3 days. DMSO (Veh) or PD0325901 containing saline was administered intraperitoneally (i.p.) at 0.5 mg/kg on days 4 and 6. The results displayed were the average and standard deviation of 3 independent experiments combined with a total of 5 mice in each condition. *p < 0.05 (paired multiple t test).

S182 on RORγt is predicted to be a target site for MAPKs (Blom et al., 2004). scRNA-seq results showed that transcripts encoding ERK2 as the most abundantly expressed MAPK across all colonic T cell subsets (Figure S6A). In human HEK293 cells transiently transfected with the FLAG-tagged RORγt expression construct, ERK inhibitor (PD0325901) treatment was sufficient to block IL-1β-mediated S182 phosphorylation on RORγt(Figure 7A). An *in vitro* assay further confirmed that recombinant ERK2 alone was sufficient to phosphorylate S182 on RORγt (Figure 7B). In cultured Th17 cells, proximity ligation assays confirmed that RORγt and ERK1/2 formed a close interaction, but their interaction was not regulated by IL-1β (Figures 7C and S6B). The inhibition of ERK (PD0325901) modestly reduced S182 phosphorylation by 15%–20% (Figure S6C), suggesting that ERKs and possibly other kinases remaining to be identified contribute to RORγt^S182^ phosphorylation in T cells.

We speculated that if specific MAPKs were involved in the phosphorylation of serine 182 in T cells, the inhibition of their kinase activities in WT cells would result in elevated IL-1R expression and cytokine productions similar to the phenotypes observed in RORγt^S182A^ cells. Consistent with this possibility, cultured WT Th17 cells treated with ERK inhibitor (PD0325901) displayed elevated cell surface IL-1R and greater IL-17A production capacity (Figures 7D and S6D). ERK inhibitor (PD0325901) treatment on RORγt^S182A^ cells, however, did not further potentiate IL-17A production, suggesting that ERK and RORγt^S182^ act on the same axis in controlling Th17 cytokine expression. We also identified that the other two ERK inhibitors (PD98059 and U0126), but not JNK (SP600125) or p38 (SB203580) inhibitors, can augment IL-17A expression (Figure S6E). Similarly, IL-10 production in cultured WT RORγt^+^ Treg-like cells generated in the presence of IL-6, IL-1β, and TGF-β (Figures S7A and S7B) were dependent on ERK and RORγt^S182^ (Figures 7E and S7C).

*In vivo*, DSS-challenged RORγt^WT^ mice treated with ERK inhibitor (PD0325901) also showed a modest increase in weight loss compared to vehicle-treated controls as expected (Figure 7F). Similar treatment on DSS-challenged RORγt^S182A^ mice, however, did not result in a more severe wasting phenotype, confirming that ERK and RORγt^S182^ in fact act on the same pathway in protecting against colonic inflammation. Together, these results demonstrate that the ERKs-RORγt^S182^ axis as a critical regulator of Th17 and RORγt^+^ Treg cell functions to protect against IL-1β-mediated inflammation.

## DISCUSSION

Recent studies reveal the importance of PTMs in modulating RORγt functions in the immune system (reviewed in Rutz et al., 2016). For example, lysine acetylations of the DBD modulate RORγt binding to chromatin DNA (Lim et al., 2015) and lysine ubiquitinations of the DBD regulate RORγt protein turnover (Kathania et al., 2016; Rutz et al., 2015). Serine phosphorylation of the LBD fine-tunes RORγt-binding partners and its transcription activities in culture Th17 cells (Chuang et al., 2018; He et al., 2017b). In this study, we report that serine 182 on the hinge region of RORγt is phosphorylated by ERKs downstream of IL-1β stimulation to maintain the proper balance and functions of Th17 and RORγt^+^ Treg cells under homeostatic and inflamed contexts.

In Th17 cells, the ERK-RORγt^S182^ pathway restricts IL-1R expression to prevent IL-17A overproduction in response to IL-1β stimulation. Other inflammatory cytokines, such as IL-6, TGF-β, and/or IL-23, do not crosstalk with the ERK-RORγt^S182^ regulatory node in Th17 cells. These results are consistent with previous reports demonstrating that ERK activation downstream of IL-1β signaling (Zhu et al., 2012) can negatively regulate Th17 effector functions (Lu et al., 2010; Tan and Lam, 2010; Zhang et al., 2015). Our findings now connect the known ERK regulatory hub to the RORγt^S182^-IL-1R axis as a key negative feedback circuit for preventing IL-1βinduced Th17 hyperactivation. In addition to Th17 cells, the IL-1β-ERK-RORγt^S182^ axis is required for maintaining a subset of the RORγt^+^ Treg populations in the steady-state colon, as well as regulating their anti-inflammatory cytokine IL-10 production capacity during colitis.

We speculate that immune subset-specific roles of RORγt^S182^ may be due to subset-specific corepressor and/or coactivator complexes interacting with the hinge region of RORγt in a cell-type and/or phosphorylation-dependent manner and should be the subject of future investigations. Additional studies will be needed to dissect whether RORγt^S182^-dependent RORγt^+^ Treg and Th17 subsets exert paracrine effects on neighboring immune cells to promote inflammation resolution during DSS and EAE. Furthermore, we suspect that the relatively modest *in vivo* effects of the ERK inhibitors administered intraperitoneally is likely due to limited bioavailability and/or rapid clearance of the drug. Future experiments using the ERK and RORγt double mutant mice will likely provide more definitive insights.

In contrast to our *in vivo* findings demonstrating that the S182 residue is dispensable for the role of RORγt in thymic T cell development and peripheral T cell differentiation, previous cell culture studies using *RORγt^−/−^* T cells retrovirally transduced with WT or mutant RORγt expression constructs reported that S182A mutant proteins interfere with thymic DP maturation and Th17 cell differentiation (He et al., 2017a, 2017b). It is important to note that T cell development is severely affected in *ROR*γ*t*−/− mice, resulting in a drastic reduction in mature T cells in circulation (Kurebayashi et al., 2000). We speculate that *ROR*γ*t*−/− T cells used in previous reports developed in the absence of RORγt are likely transcriptionally and functionally distinct from T cells developed via the canonical RORγt-dependent mechanism. In addition, these previous reports used IL-6 and TGF-β to polarize virally transduced *ROR*γ*t*^−/−^ T cells toward the Th17 lineage *in vitro*. Based on results from our *in vitro* and *in vivo* experiments, IL-1β, but not IL-6 and TGF-β, is necessary for engaging the ERK-RORγt^S182^ circuit. Therefore, the RORγt^S182A^ mouse model reported here provides an excellent opportunity for uncoupling the developmental and differentiation roles of RORγt from its immune effector roles in mature T cells, advancing efforts to understand the extent to which RORγt contributes to tissue homeostasis *in vivo*. Given the important roles of Th17 and RORγt^+^ Treg cells in settings of numerous autoimmune diseases (Guo, 2016; Martinez et al., 2014; Xu et al., 2018), we speculate that the development of pharmacological strategies that can activate the IL-1β-ERK-RORγt^S182^ negative feedback circuit and restore Th17-RORγt^+^ Treg balance will provide a potential opportunity for combating T cell-mediated inflammatory diseases.

### Limitations of the study

We have not excluded the possible contribution(s) of additional RORγt-expressing cells, including Tγδ17, ILC3, and neutrophils, to the DSS and EAE phenotypes observed in the RORγt^S182A^ total knockin mice. We have not assessed the relative and/or temporal contributions of RORγt^S182A^ Th17 hyperactivation and reduced the suppressive functions of RORγt^S182A^ RORγt^+^ Treg to elucidate whether one or both alterations underlie the exacerbated inflammatory phenotypes observed. The *in vitro* T cell polarization conditions described in this study generated a mix culture of 20%–30% RORγt^+^ Treg-like cells and 70%–80% Th17. Although this ratio reflected those found in the intestine *in vivo*, further culture condition optimization will be needed to yield homogeneous RORγt^+^ Treg-like cells for biochemical studies to better address whether phosphorylated RORγt regulates its targets directly or indirectly. We have not performed extensive protein-level validation to confirm results from our scRNA-seq analysis. If cluster specific gene expression differences can be confirmed at the protein level, then we speculate that cluster-specific cell-surface molecules can be helpful markers for identifying and capturing specific *in vivo* T cell subsets for future mechanistic studies.

## STAR★METHODS

### RESOURCE AVAILABILITY

#### Lead contact

Further information and requests for resources and reagents should be directed to and will be fulfilled by the lead contact, Wendy Jia Men Huang (wendyjmhuang@ucsd.edu).

#### Materials availability

Plasmids and primer sequences in this study will be available upon request. Mouse line RORγt^S182A^ on C57BL/6 background generated in this study will be available upon request and with approval of institutional Material Transfer Agreements (MTA).

#### Data and code availability

- Single-cell RNA-seq data have been deposited at GEO and are publicly available as of the date of publication. Accession numbers are listed in the key resources table. Original western blot images and microscopy data will be shared by the lead contact upon request. This paper also analyzes an existing, publicly available data. Accession number for this dataset is listed in the key resources table.
- This paper does not report original code.
- Any additional information required to reanalyze the data reported in this paper is available from the lead contact upon request.
- The accession numbers for the scRNA-seq data reported in this paper are available on GEO: GSE173887.

### EXPERIMENTAL MODEL AND SUBJECT DETAILS

For *in vivo* animal studies, adult RORγt^WT^ and homogenous knock-in (RORγt^S182A^) cohoused littermates (both male and female in equal proportion) on C57BL/6J background between eight to twelve weeks old were used. Littermates of the same sex were randomly assigned to experimental groups. Our vivarium at UCSan Diego is kept under specific pathogen free (SPF) conditions. Regular serology and PCR tests ensure the absence of Epizootic diarrhea of infant mice (rotavirus, EDIM), Mouse hepatitis virus (MHV), Mouse parvovirus (MPV), Minute virus of mice (MVM), Theiler’s murine encephalomyelitis virus (TMEV), fur mites and pinworms. All animal studies were approved and followed the Institutional Animal Care and Use Guidelines of the University of California San Diego.

Primary T cell cultures were obtained from adult RORγt^WT^ and homogenous knock-in (RORγt^S182A^) cohoused littermates (both male and female in equal proportion) on C57BL/6J background between eight to twelve weeks old. Mouse naive T cells were purified from spleens and lymph nodes using the Naive CD4^+^ T Cell Isolation Kit according to the manufacturer’s instructions (Miltenyi Biotec). Cells were cultured in Iscove’s Modified Dulbecco’s Medium (IMDM, Sigma Aldrich) supplemented with 10% heat-inactivated FBS (Peak Serum), 50U/50 ug penicillin-streptomycin (Life Technologies), 2 mM glutamine (Life Technologies), and 50 μM β-mercaptoethanol (Sigma Aldrich). For Th17 cell polarization, naive cells were seeded in 24-well or 96-well plates, pre-coated with rabbit anti-hamster IgG, and cultured in the presence of 0.25μg/mL anti-CD3ε (eBioscience), 1μg/mL anti-CD28 (eBioscience), 20ng/mL IL-6 (R&D Systems) with the addition of 0.3ng/mL TGFβ (R&D Systems), and/or 20ng/mL IL-1b (R&D Systems), and/or 25ng/mL IL-23 (R&D Systems) for 72 hours. For generating RORγt^+^ Treg-like cells in culture, naive cells were cultured in the presence of 0.25μg/mL anti-CD3ε (eBioscience), 1μg/mL anti-CD28 (eBioscience), 20ng/mL IL-6 (R&D Systems), 5ng/mL TGFβ (R&D Systems), and 20ng/mL IL-1 β (R&D Systems) for 72 hours. Inhibitors of JNK (SP600125, 10μM), p38 (SB203580, 10μM), or ERK inhibitors (PD98059, 10μM; PD0325901, 5μM and U0126, 5μM) were obtained from MedChemExpress. Cultured cells were treated at 48 hours after polarization. For co-culture experiments, naïve CD4^+^ cells were activated with 0.25μg/mL anti-CD3ε (eBioscience), 1μg/mL anti-CD28 (eBioscience) overnight, and then transduced with either NGFR or Thy1.1 expressing constructs, mixed in 1:1 ratio and cocultured as described above.

### METHOD DETAILS

#### DSS induced colitis

Dextran Sulfate Sodium Salt (DSS) Colitis Grade 36,000-59,000MW (MP Biomedicals) was added to the drinking water at a final concentration of 2% (wt/vol) and administered for 7 days. Mice were weighed every other day. On day 10, colons were collected for histology staining and lamina propria cells were isolation as described (Lefrancois and Lycke, 2001). Cells were kept for RNA isolation or flow cytometry. The colonic sections from mice were scored in a double-blind fashion as described previously (Abbasi et al., 2020; Koelink et al., 2018).

#### T cell transfer colitis

For T cell transfer model of colitis, 0.5 million naive CD4^+^ T cells were isolated from mouse splenocytes using the Naive CD4^+^ T Cell Isolation Kit (Miltenyi), and administered to *Rag1*^−/−^ recipients intraperitoneally. For co-transfer experiments, CD4^+^ T cells from RORγt^WT^ and RORγt^S182A^ mice were transduced with GFP expressing construct (pMIG) or Thy1.1 expressing construct (MSCV), mixed in a 1:1 ratio, and i.p. into RAG1^−/−^ mice. Weights were monitored twice a week for a total of 33 days.

#### EAE model

EAE was induced in 8-week-old mice by subcutaneous immunization with 100 μg myelin oligodendrocyte glycoprotein (MOG35–55) peptide (GenScript Biotech) emulsified in complete Freund’s adjuvant (CFA, Sigma-Aldrich), followed by administration of 400 ng pertussis toxin (PTX, Sigma-Aldrich) on days 0 and 2 as described (Bittner et al., 2014). Clinical signs of EAE were assessed as follows: 0, no clinical signs; 1, partially limp tail; 2, paralyzed tail; 3, hind limb paresis; 4, one hind limb paralyzed; 5, both hind limbs paralyzed; 6, hind limbs paralyzed, weakness in forelimbs; 7, hind limbs paralyzed, one forelimb paralyzed; 8, hind limb paralyzed, both forelimbs paralyzed; 9, moribund; 10, death.

#### Flow cytometry

Colonic lamina propria cells were digested with Dispase (Worthington), Dnase I (Millipore Sigma) and Collagenase D (Roche), and isolated with Percoll (GE healthcare). Spinal cords were dissected and digested with Collagenase D; immune cells were isolated with 38% Percoll. Cells were stimulated with 5 ng/mL Phorbol 12-myristate 13-acetate (PMA, Millipore Sigma) and 500ng/mL ionomycin (Millipore Sigma) in the presence of GoligiStop (BD Bioscience) for 5 hours at 37°C, followed by cell surface marker staining. Fixation/Permeabilization buffers (eBioscience) were used per manufacturer instructions to assess intracellular transcription factor and cytokine expression. Antibodies are listed in key resources table.

#### scRNA-seq and analysis

Colonic lamina propria cells from control or DSS treated mice were collected and enriched for CD4^+^ T cells using the mouse CD4^+^ T cell Isolation Kit (Miltenyi). Enriched CD4^+^ cells (~10,000 per mouse) were prepared for single cell libraries using the Chromium Single Cell 3’ Reagent Kit (10xGenomics). The pooled libraries of each sample (20,000 reads/cell) were sequenced on Nova-Seq S4 following the manufacturer’s recommendations. Cellranger v3.1.0 was used to filter, align, and count reads mapped to the mouse reference genome (mm10-3.0.0). The Unique Molecular Identifiers (UMI) count matrix obtained was used for downstream analysis using Seurat (v4.0.1) (Hao et al., 2021). The cells with mitochondrial counts >5%, as well as outlier cells in the top and bottom 0.2% of the total gene number detected index were excluded. After filtering, randomly selected 10,000 cells per sample were chosen for downstream analysis. Cells with *Cd4* expression lower than 0.4 were removed, resulting in 27,420 total cells from eight samples. These cells were scaled and normalized using log-transformation, and the top 3,000 genes were selected for principal component analysis. The dimensions determined from PCA were used for clustering and non-linear dimensional reduction visualizations (UMAP). Differentially expressed genes identified by FindMarkers were used to characterize each cell cluster. Other visualization methods from Seurat such as VlnPlot, FeaturePlot, and DimPlot were also used. The accession numbers for the scRNA-seq data reported in this paper are available on GEO: GSE173887.

#### FLAG tag pulldown and *in vitro* phosphorylation assay

HEK (293) cells were seeded in 10 cm tissue culture plates at a density of 4 million cells per plate in 8mL of DMEM containing 10% FBS, and then incubated overnight at 37° C. 2mg of plasmid DNA (pcDNA or pcDNA-2xFLAG-RORγt) was transfected into cells using 6mL of X-tremeGENE™ HP DNA Transfection Reagent (Roche) at 37°C for 16hrs in 8mL of DMEM containing 1% FBS for serum starvation, followed by stimulation with 10ng/mL hIL-1β (R&D) and/or hIL-23 (R&D). The treatment of 5 mM PD0325901 was performed at the same time as the serum starvation. Whole cell lysates (WLC) were prepared as previously described (Abe et al., 2018) with modifications as follows. HEK (293) cells were sonicated in cell lysis buffer (50mM HEPES-KOH (pH 7.9), 150mM NaCl, 1.5mM MgCl_2_, 1% NP-40, 1mM Na_3_VO_4_, 1mM PMSF (Sigma-Aldrich), 1X protease inhibitor cocktail (Sigma-Aldrich)) by ultrasound homogenizer (Bioruptor, Diagenode) for 10min at 4°C. For immunoblotting of input samples, aliquots of WCL were boiled at 95°C for 5min in NuPAGETM LDS Sample Buffer (Thermo Fisher Scientific) with NuPAGE™ Sample Reducing Agent (Thermo Fisher Scientific), subjected to SDS-PAGE, and transferred to immobilon-P transfer membranes (Merck Millipore). Immunodetection was carried out with the indicated antibodies (Table S1) and bound antibodies were visualized with peroxidase-conjugated affinity-purified donkey anti-mouse or anti-rabbit IgG (Dako) using LuminateTM Forte Western HRP Substrate (Merck Millipore), and luminescence images were analyzed by ChemiDoc XRS+ System (Bio-Rad Laboratories). For FLAG tag pulldown, WCL were immunoprecipitated in cell lysis buffer by wheel rotating for 4hrs at 4°C in the presence of anti-FLAG M2 affinity gel (Sigma-Aldrich). For *in vitro* phosphorylation assay, the immunoprecipitated affinity gel was washed three times with cell lysis buffer and three times with PBS, and then incubated with 0.5mg of recombinant ERK2 (EMD Millipore, 14-173) in 60mL of reaction buffer (50mM Tris-HCl (pH 7.5), 0.1mM EGTA, 0.1mM EGTA, 0.1mM Na_3_VO_4_, 0.1% 2-Mercaptoethanol, 1mg/mL BSA, 0.1mM ATP, 15mM MgCl_2_) for 1hr at 30°C. After washed three times with PBS, the affinity gel was incubated with 150ng/mL 3X FLAG peptide (Sigma-Aldrich) for 30 min at 4°C. The elution was subjected to immunoblotting as described above.

#### Proximity ligation assay

Proximity ligation assays on cultured Th17 cells were performed in accordance with the Duolink® PLA Fluorescence Protocol (Sigma-Aldrich). Briefly, cells were fixed with 3.7% formaldehyde on coverslips and permeabilized with 0.1% Triton X-100, followed by blocking, hybridization with primary antibodies, incubation with PLA probes, and lastly the ligation-amplification steps. Coverslips mounted in DAPI-containing medium and imaged on the Leica SP8 confocal microscope (63x Oil objective, DAPI and Alex 594 channels, room temperature) at the UCSD School of Medicine Microscopy Core. Automated fluorescent particle analysis was performed by ImageJ/Fiji software where the threshold was set to remove background fluorescent and segment individual particles as described (Debaize et al., 2017).

#### cDNA synthesis, qRT-PCR, and RT-PCR

Total RNA was extracted with the RNeasy kit (QIAGEN) and reverse transcribed using iScript™ Select cDNA Synthesis Kit (Bio-Rad Laboratories). Real time RT-PCR was performed using iTaq™ Universal SYBR® Green Supermix (Bio-Rad Laboratories). Expression data was normalized to *Gapdh* mRNA levels.

### QUANTIFICATION AND STATISTICAL ANALYSIS

Statistical details of each experiment can be found in the figure legends, including the statistical tests used, exact number of animals (n) used, and the number of times each assay was performed. All values are presented as means ± standard deviation. Significant differences were evaluated using GraphPad Prism 8. The student’s t-test, paired t-test, or multiple t-tests were used to determine significant differences. A two-tailed p-value of <0.05 was considered statistically significant in all experiments.

## Supplementary Material

1

## Figures and Tables

**Table:** KEY RESOURCES TABLE

REAGENT or RESOURCE	SOURCE	IDENTIFIER
Antibodies		
RORg pS203 / RORgt pS182	Rockland-Inc	Cat# 600-401-GR8
Bcl-xL (54H6), Alexa Fluor™ 488 conjugated	Cell Signaling	Cat# 2767S; RRID: AB_2274763
RORgt (B2D), PE-eFluor 610 conjugated	Thermo Fisher Scientific	Cat# 61-6981-82; RRID: AB_2574650
IL-10 (JES5-16E3), PE conjugated	Thermo Fisher Scientific	Cat# 12-7101-41; RRID: AB_466174
FOXP3 (FJK-16s), PE-Cyanine5.5 conjugated	Thermo Fisher Scientific	Cat# 35-5773-82; RRID: AB_11218094
CD3e (eBio500A2), APC-eFluor 780 conjugated	Thermo Fisher Scientific	Cat# 47-0033-82; RRID: AB_2637316
CD11c (N418), PE-Cyanine5.5 conjugated	Thermo Fisher Scientific	Cat# 35-0114-82; RRID: AB_469709
IL-17A (TC11-18H10.1), APC/Cyanine7 conjugated	BioLegend	Cat# 506939; RRID: AB_2565780
IL-17F (9D3.1C8), Alexa Fluor™ 488 conjugated	BioLegend	Cat# 517006; RRID: AB_10661903
CD4 (GK1.5), PE/Cyanine5 conjugated	BioLegend	Cat# 100410; RRID: AB_312695
CD8a (53-6.7), Alexa Fluor™ 488 conjugated	BioLegend	Cat# 100723; RRID: AB_389304
TCR gamma/delta (UC7-13D5), FITC conjugated	BioLegend	Cat# 107503; RRID: AB_313312
CD121a, IL-1 R, Type I/p80 (JAMA-147), PE conjugated	BioLegend	Cat# 113505; RRID: AB_2125036
F4/80 (BM8), PE conjugated	BioLegend	Cat# 123109; RRID: AB_893498
CD11b (M1/70), APC/Cyanine7 conjugated	BioLegend	Cat# 101226; RRID: AB_830642
CD3e (145-2C11), Functional Grade	Thermo Fisher Scientific	Cat# 16-0031-86, RRID: AB_468849
CD28 (37.51), Functional Grade	Thermo Fisher Scientific	Cat# 16-0281-86, RRID: AB_468923
p44/42 MAP Kinase	Cell Signaling	Cat# 4696, RRID: AB_390780
ANTI-FLAG® M2 Affinity Gel	Sigma-Aldrich	Cat# A2220, RRID: AB_10063035
Chemicals, peptides, and recombinant proteins		
SP600125	MedChemExpress	Cat# HY-12041
SB203580	MedChemExpress	Cat# HY-10256
PD98059	MedChemExpress	Cat# HY-12028
PD0325901	APExBIO	Cat# A3013
U0126	MedChemExpress	Cat# HY-12031
Recombinant Human TGF-beta 1 Protein	R&D	Cat# 240-B
Recombinant Mouse IL-6 Protein	R&D	Cat# 406-ML
Recombinant Mouse IL-1 β Protein	R&D	Cat# 401-ML
Recombinant Mouse IL-23 Protein	R&D	Cat# 1887-ML
MAP Kinase 2/Erk2 Protein, active, mouse	Millipore Sigma	Cat# 14-173
Recombinant Human IL-23 Protein	R&D	Cat# 1290-IL
Recombinant Human IL-1 β Protein	R&D	Cat# 201-LB
Critical commercial assays		
Chromium Single Cell 3’ Reagent Kit	10xGenomics	PN-1000121
Naive CD4^+^T Cell Isolation Kit, mouse	Miltenyi Biotec	Cat# 130-104-453
Duolink® PLA Starter Kits	Millipore Sigma	DUO92101
Deposited data		
Single cell RNA-seq	This paper	GEO: GSE173887
Experimental models: Cell lines		
Human: Platinum-E (Plat-E) Retroviral Packaging Cell Line	Cell Biolabs, Inc.	Cat# RV-101
Human: HEK293	ATCC	CRL-1573
Experimental models: Organisms/strains		
Mouse: RORγt^S182A^, C57BL/6	This paper	N/A
Mouse: B6.129S7-*Rag1^tm1Mom^*/J	The Jackson Laboratory	Strain #002216
Oligonucleotides		
Primers for qPCR, see Table S2	This paper	N/A
Recombinant DNA		
MSCV-IRES-GFP (pMIG)	Addgene	RRID: Addgene_9044
MSCV-IRES-NGFR	Addgene	RRID: Addgene_27489
MSCV-IRES-Thy1.1	Addgene	RRID: Addgene_17442
MSCV-mRorgt(wt)-IRES-Thy1.1	(Huh et al., 2011)	N/A
MSCV-mRorgt(S182A)-IRES-Thy1.1	This paper	N/A
MSCV-mRorgt(S182D)-IRES-Thy1.1	This paper	N/A
pcDNA3.1	Invitrogen	Cat# V79020
pcDNA3.1-mRorgt(wt)-Cterm2xFLAG	This paper	N/A
Software and algorithms		
ImageJ	National Institutes of Health, Bethesda, MD, USA	https://imagej.nih.gov/ij/
Cellranger v3.1.0	10xGenomics	https://support.10xgenomics.com/single-cell-gene-expression/software/pipelines/latest/installation
Seurat (v4.0.1)	(Hao et al., 2021)	https://satijalab.org/seurat/
Prism 8	GraphPad	https://www.graphpad.com/scientific-software/prism/
Other		
Sequence data, analyses, and resources related to RORγt^−/−^ cultured Th17 cells	(Ciofani et al., 2012)	GEO: GSE40918

## Highlights

- The master T cell transcription factor, RORγt, is phosphorylated on S182 by ERK2
- IL-1β activates the ERK-RORγt axis to modulate Th17 and RORγt^+^ Treg functions
- Phospho-null mice experience exacerbated tissue inflammation in disease models
- The ERK2-RORγt axis is dispensable for normal thymic T cell development
