# Supplementary material for: RORγt phosphorylation protects against T cell-mediated inflammation
Source: Cell Rep. Author manuscript; Available in PMC 2022 Apr 5. (PMC8982147; doi:10.1016/j.celrep.2022.110520)
Supplement: 1 [file NIHMS1789670-supplement-1.pdf]

Cell Reports, Volume *38*

Supplemental information

RORγt phosphorylation protects against T cell-mediated inflammation

Shengyun Ma, Shefali A. Patel, Yohei Abe, Nicholas Chen, Parth R. Patel, Benjamin S. Cho, Nazia Abbasi, Suling Zeng, Bernd Schnabl, John T. Chang, and Wendy Jia Men Huang

Supplemental information

Figure S1. RORγt^{S182} is dispensable for normal thymic T cell development *in vivo.* Related to Figure 1.

A. Top: position of major post-translationally modified sites on RORγt. Phosphorylation (p), methylation (m). Bottom: representative mass spectral map of one murine RORγt peptide carrying a phosphorylated S182 residue.

B. CRISPR-Cas9 mediated genomic mutations to generate the RORγt^{S182A} knock-in mice.

C. Representative flow cytometry analysis of CD4 and CD8α expression on thymocytes. This experiment was repeated three times on independent biological samples with similar results.

D. RORγt gMFI in thymic DP cells (CD4^{+}CD8α^{+}) from RORγt^{WT} (n=3) and RORγt^{S182A} (n=3) cohoused littermates. Each dot represents result from one mouse. Each bar represents sample mean. n.s. not significant (t-test).

E. Representative histogram of Bcl-xL expression in thymic DP cells from a pair of RORγt^{WT} (black) and RORγt^{S182A} (brown) littermates. This experiment was repeated three times on independent biological samples with similar results. Isotype control shown in grey.

Figure S2. Steady state RORγt^{+} T cell subsets in the intestine. Related to Figure 1.

A. Proportion of Th17 (CD4^{+}RORγt^{+}Foxp3^{-}), RORγt^{+} Treg (CD4^{+}RORγt^{+}Foxp3^{+}), conventional Treg (CD4^{+}RORγt^{-}Foxp3^{+}), Tγδ17 (TCRγδ^{+}RORγt^{+}), and type 3 innate lymphoid cells (ILC3: CD3ε^{-}RORγt^{+}Foxp3^{-}) in the steady state small intestinal

lamina propria of RORγt^{WT} (n=6) and RORγt^{S182A} (n=6) cohoused littermates. Each dot represents result from one mouse. Each bar represents sample mean. n.s. not significant (t-test).

B. Proportion of Th17, RORγt^{+} Treg, conventional Treg, Tγδ17 and ILC3 cells in the steady state colonic lamina propria of RORγt^{WT} (n=6) and RORγt^{S182A} (n=6) cohoused littermates. Each dot represents result from one mouse. Each bar represents sample mean. n.s. not significant (t-test).

Figure S3. scRNA-seq to characterize colonic T cell subsets. Related to Figure 1.

A. RNA expression level of *Il17a* and *Il10* in total colonic lamina propria (cLP) cell lysate from steady state mice. Each dot represents result from one mouse. Each bar represents sample mean. n.s. not significant (t-test).

B. Proportion of IL-17A^{+} (up) or IL-10^{+} (bottom) Th17, RORγt^{+} Treg, and conventional Treg cells in the steady state colon. Each dot represents result from one mouse. Each bar represents sample mean. n.s. not significant (multiple t-test).

C. scRNA-seq experiment workflow: colonic lamina propria CD4^{+} cells were enriched with anti-CD4 microbeads. Transcriptome profiles of the captured cells were revealed using the 10x Genomics droplet-based 3′ scRNA-seq approach. UMAP plots of the twelve immune cell clusters obtained (bottom, left) and the average expression of *Cd3e* (bottom, right).

D. Heatmap of mean scaled average expression of subset enriched genes from the indicated *Cd4*$^{+}$*Cd3e*$^{+}$ clusters.

E. Top: closed up UMAP plot showing two neighboring populations of colonic Th17 cells in red (cluster 0) and green (cluster 3). Bottom: select expressions of transcripts encoding the indicated cell surface receptors and effector molecules differentially enriched in the two Th17 subsets.
F. Heatmap of mean scaled average expression of subset enriched genes from cluster

Figure S4. Colonic RORγt^{+} immune cells from DSS-challenged mice. Related to Figure 2 and 3.

A. RNA expression level of *Il17a* and *Il10* in total cLP cell lysate from steady state mice. Each dot represents result from one mouse. Each bar represents sample mean. n.s. not significant (t-test).
B. Heatmap of mean scaled Log_2 fold change of RORγt^{S182}-dependent Th17 genes from steady state and DSS challenged colonic lamina propria.
C. Representative flow analysis RORγt and Foxp3 in colonic lamina propria CD4^{+} T cells from DSS-challenged mice.
D. RORγt gMFI in colonic RORγt^{WT} and RORγt^{S182A} CD4^{+} cells from DSS-challenged mice. Each dot represents result from one mouse. Each bar represents sample mean. n.s. not significant (t-test).

Figure S5. IL-17A production potential of colonic and splenic RORγt^{+} cells from RAG1$^{-/-}$ mice. Related to Figure 4A.

A. Cell number of total and IL-17A producing colonic Th17, RORγt^{+} Treg, and Treg cells from DSS-challenged RORγt^{WT} and RORγt^{S182A} mice. Each dot represents

result from one mouse. Each bar represents sample mean. * p-value<0.05, n.s. not significant (multiple t-test, n=4).

B. Cell number of total and IL-17A producing splenic Th17, RORγt^{+} Treg, and Treg cells from DSS-challenged RORγt^{WT} and RORγt^{S182A} mice. Each dot represents result from one mouse. Each bar represents sample mean. * p-value<0.05, n.s. not significant (multiple t-test, n=4).

Figure S6. ERK-RORγt^{S182} axis regulates IL-17A production potential in culture Th17 cells. Related to Figure 7.

A. Heatmap of average expression of various MAPKs in the indicated colonic T cell clusters as determined by scRNA-seq.

B. Proximity ligation assay (PLA) signals of RORγt and ERK1/2 in RORγt^{WT} Th17 cells cultured in the indicated conditions. Each dot represents result from one cell.

C. IB quantification of WCL from RORγt^{WT} Th17 cells treated with dimethylsulfoxide (DMSO, Veh) or ERK inhibitor (PD0325901, 5μM). Relative abundance: p-S182 signals were normalized to total RORγt signals. Each dot represent result from one independent experiment.

D. Proportion of IL-17A^{+}, IL-1R^{high}, or IL-23R^{high} among Th17 cells cultured in IL-6 (20ng/ml), IL-1β (20ng/ml), IL-23 (25ng/ml) and treated with vehicle (DMSO) or ERK inhibitor (PD0325901, 5μM) at 48hrs. Cells were harvested at 72hrs. Each dot represents result from one mouse. Each bar represents sample mean. * p-value<0.05, n.s. not significant (unpaired t-test).

E. Proportion of IL-17A$^+$ among Th17 cells cultured in IL-6 (20ng/ml), IL-1β (20ng/ml), IL-23 (25ng/ml) and treated with vehicle (DMSO) or the indicated inhibitors (JNK^{inh}: SP600125, 10 μM; P38^{inh}: SP203580, 10 μM, ERK^{inh}: PD98059, 10 μM or U0126, 5 μM)) at 48hrs. Cells were harvested at 72hrs. Each dot represents result from one mouse. Each bar represents sample mean.

Figure S7. *In vitro* polarization of naïve CD4$^+$ T cells to generate mixed Th17, RORγt$^+$ Treg, and Treg-like cultures. Related to Figure 7.

A. Representative flow cytometry analysis of RORγt and Foxp3 in CD4$^+$ RORγt^{WT} cells cultured in the indicated conditions.
B. Proportion of IL-10$^+$ producers from cells cultured in the presence of IL-6 (20ng/ml), IL-1β (20ng/ml), and TGFβ (5ng/ml). SSC: side scatter.
C. Left: workflow of the co-culture experiment using the indicated transduced and marked RORγt^{WT} and RORγt^{S182A} CD4$^+$ T cells polarized in the presence of IL-6 (20ng/ml), IL-1β (20ng/ml), and TGFβ (5ng/ml). Right: summarized proportion of IL-10$^+$ in cultured cells 3 days post polarization. Each line represents results from one experimental well. Each bar represents mean from 8 independent experiments. **** p-value<0.0001 (paired t-test).

Figure S1. RORγtS182 is dispensable for normal thymic T cell development *in vivo*.

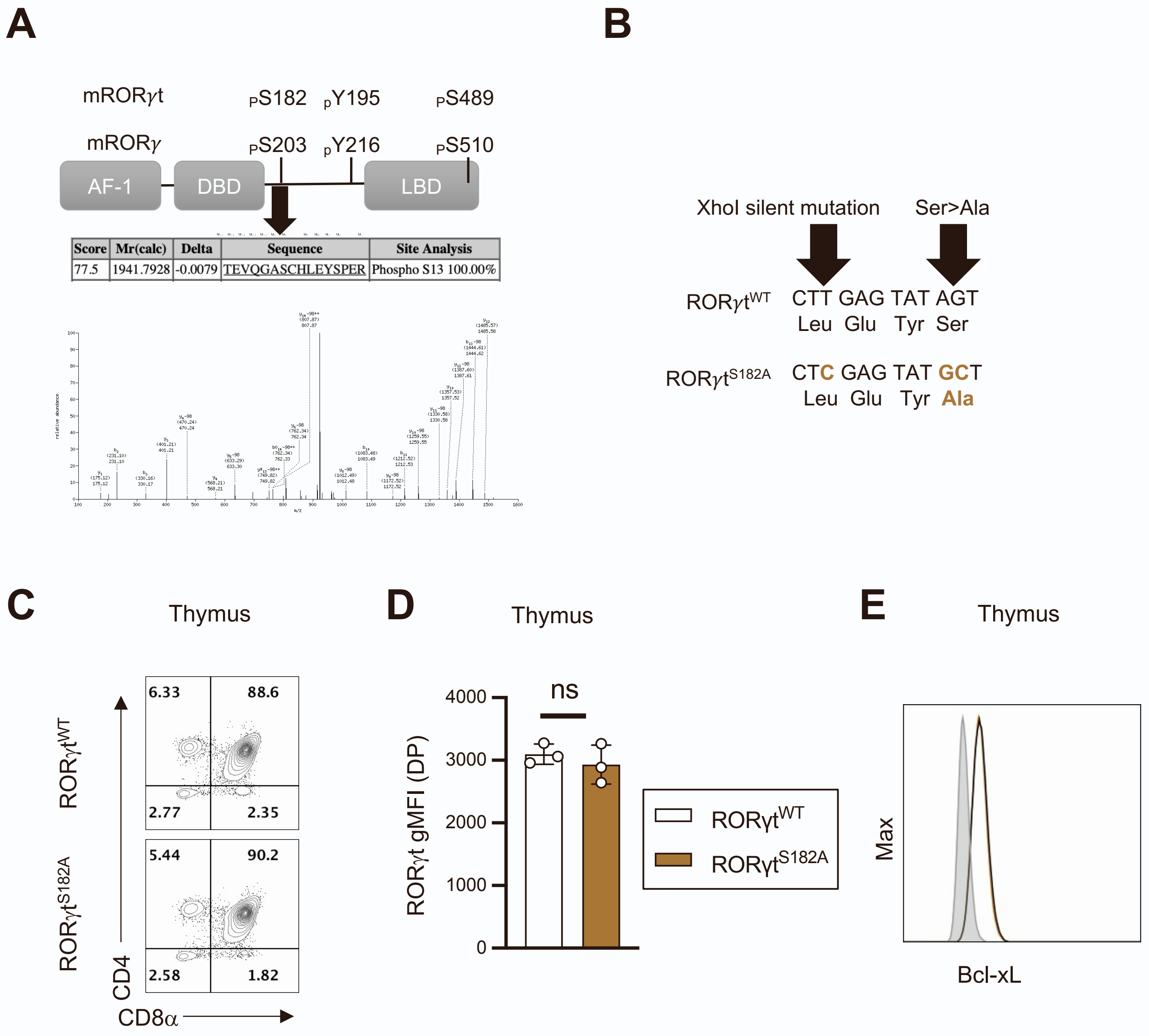

Figure S2. Steady state RORγt⁺ immune subsets in the intestine.

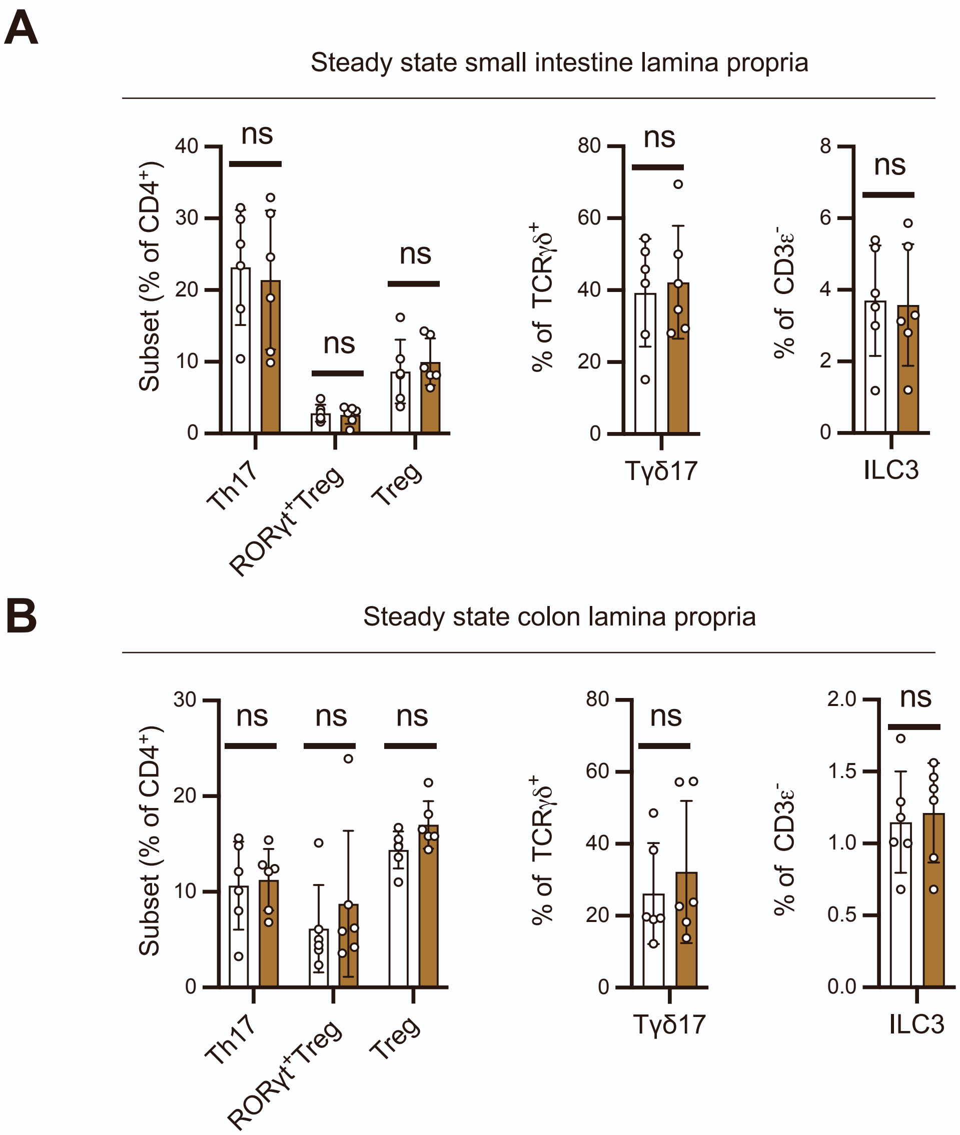

Figure S3. scRNA-seq to characterize colonic T cell subsets.

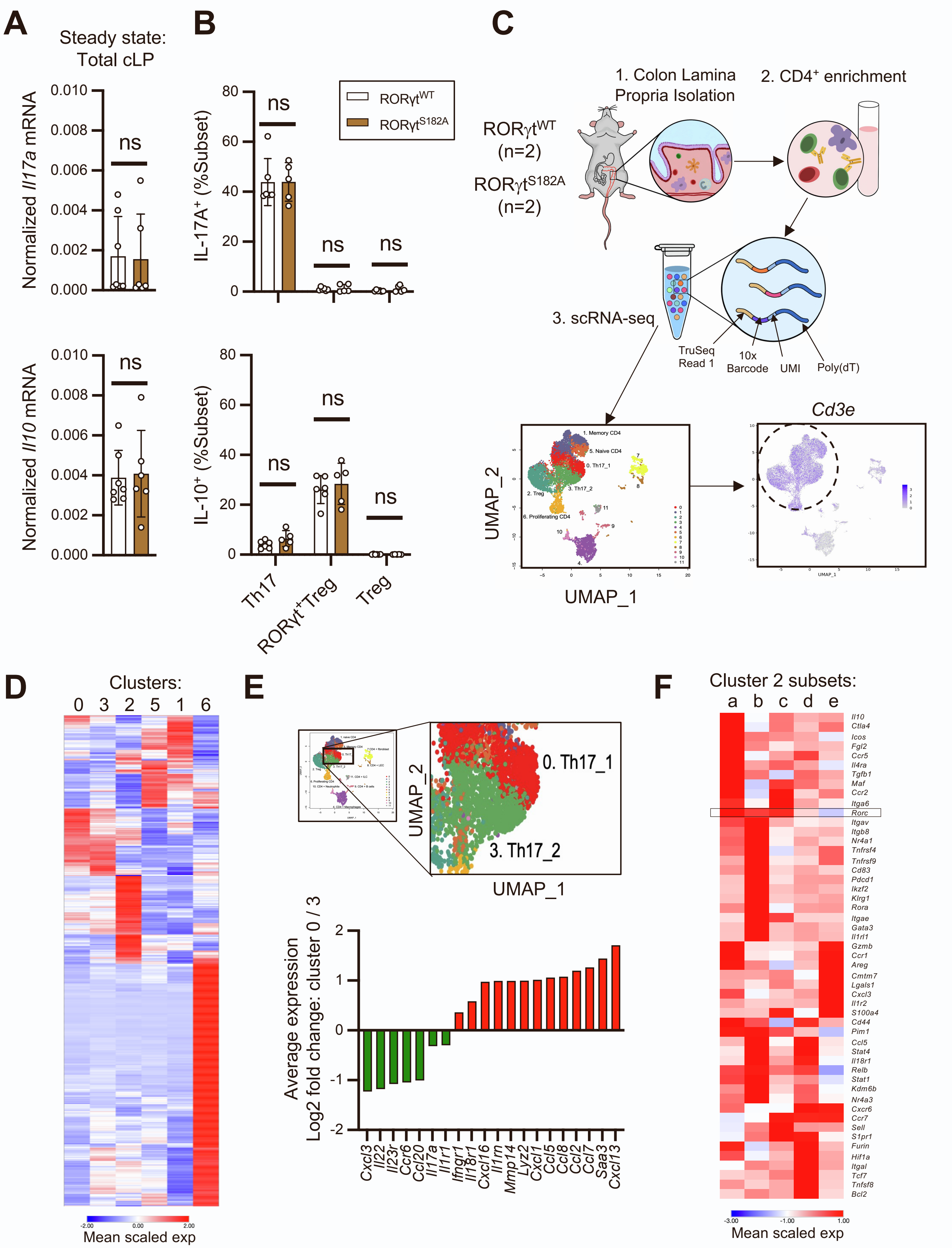

Figure S4. Colonic RORγt⁺ immune cells from DSS-challenged mice.

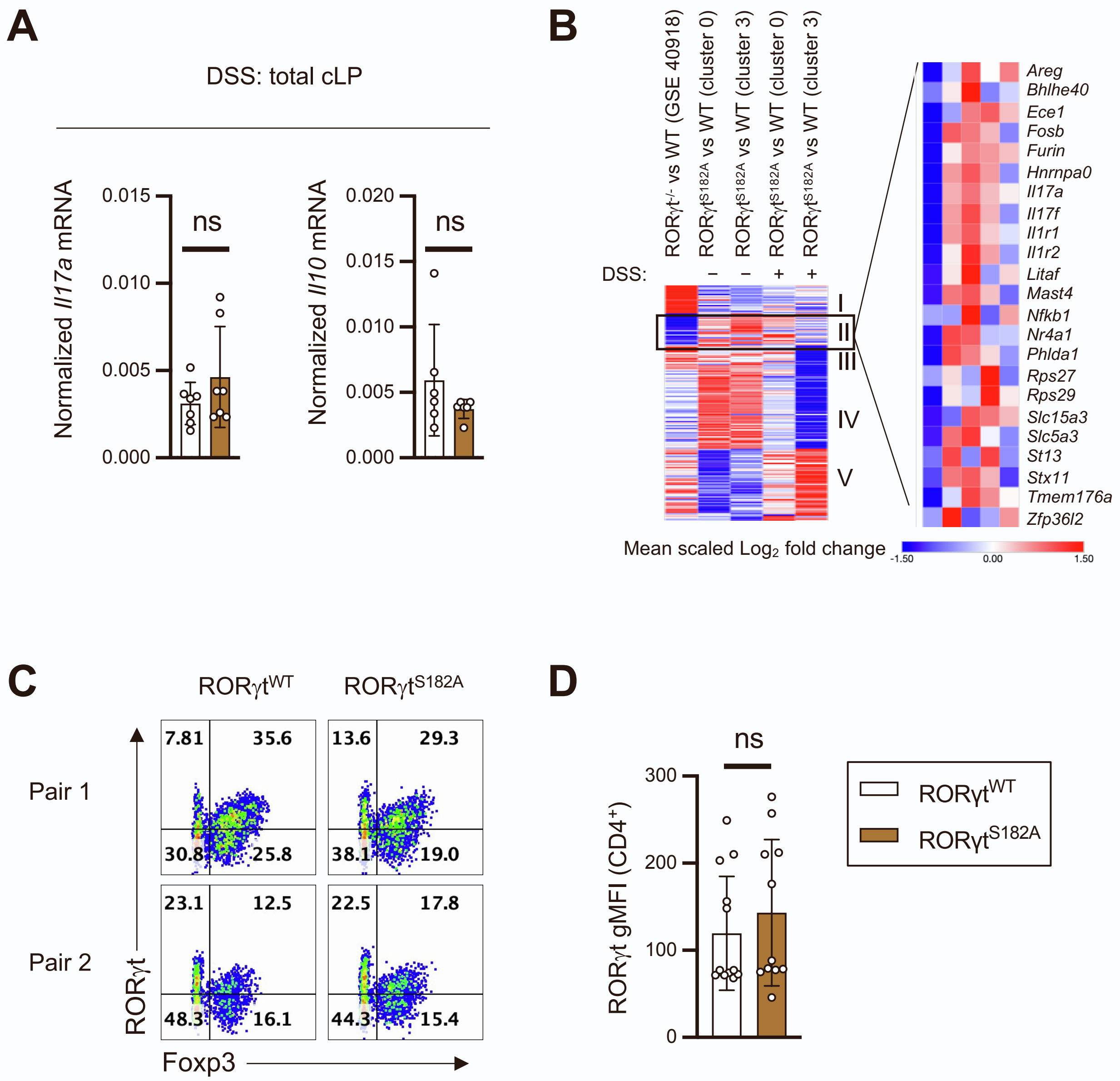

Figure S5. IL-17A production potential of colonic and splenic RORγt⁺ cells from RAG1⁻/⁻ mice.

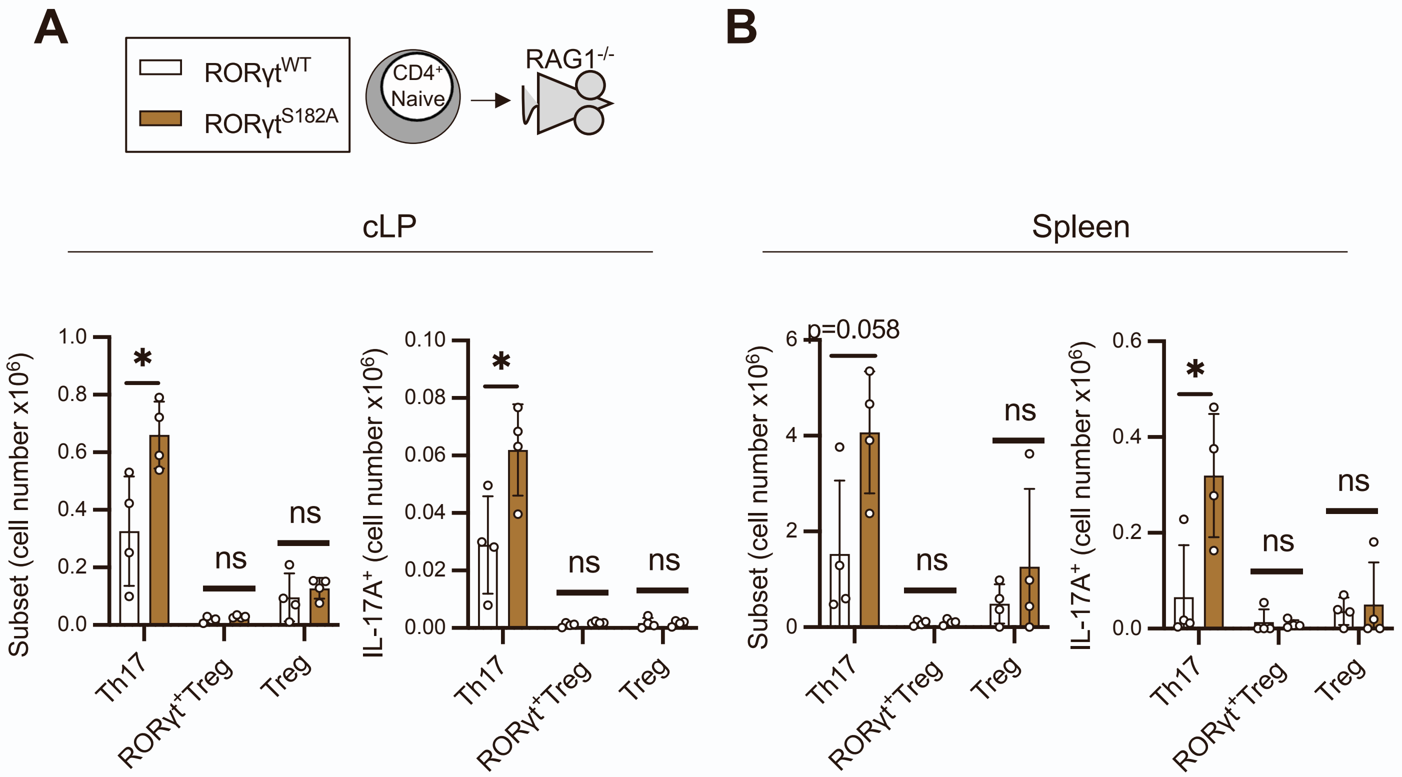

Figure S6. ERK-RORγt^{S182} axis regulate IL-17A production potential in cultured Th17 cells.

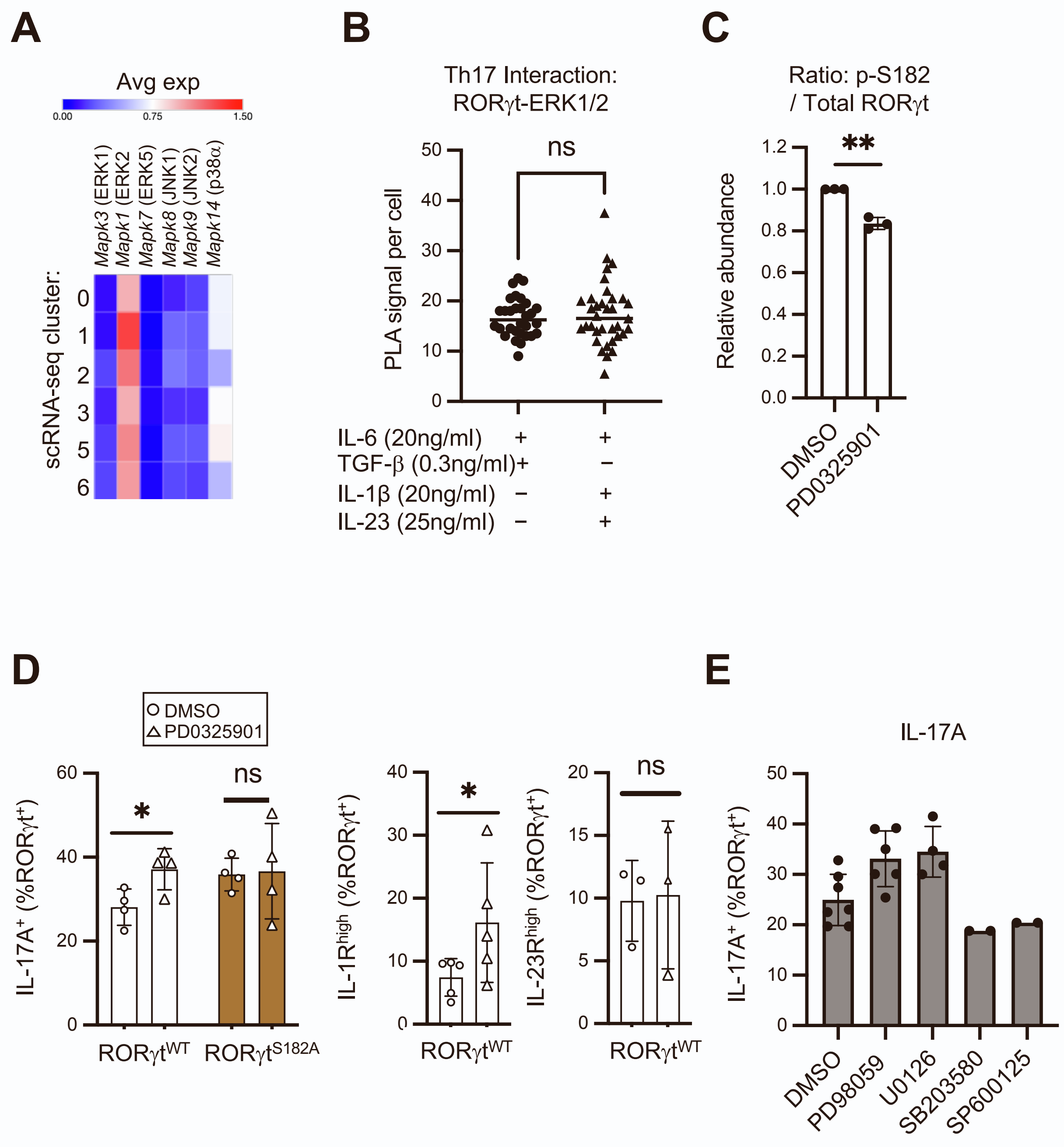

Figure S7. *In vitro* polarization of naïve CD4$^+$ T cells to generate mixed Th17, RORγt$^+$ Treg, and Treg-like cultures.

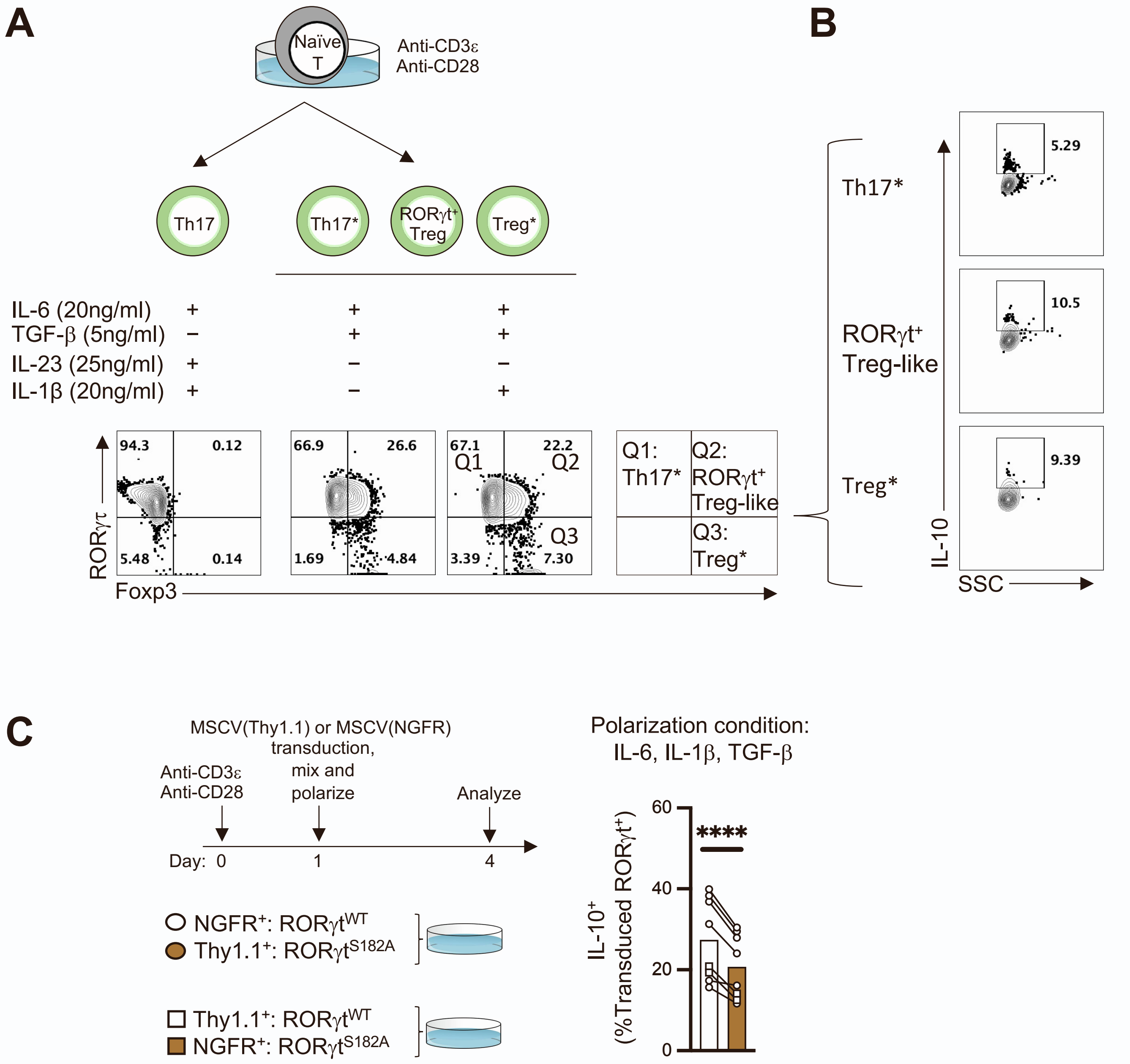

Table S1. Antibody information. Related to Figure 1-7.

Name	Catalog number	Company	Application
RORg pS203 / RORgt pS182	600-401-GR8	Rockland-Inc	WB
FLAG	F1804	Sigma-Aldrich	WB
β-Actin	A2228	Sigma-Aldrich	WB
RORγt		Covance	Proximity Ligation Assay
BCL-xL	2767S	Cell Signaling	Flow cytometry
ERK1/2	4696	Cell Signaling	Proximity Ligation Assay
CD11b	101226	BioLegend	Flow cytometry
CD11c	15-1171-81	eBiosciences	Flow cytometry
CD121a/IL1R	113505	BioLegend	Flow cytometry
CD3ε	147-0033-82	eBiosciences	Flow cytometry
CD4	100410	BioLegend	Flow cytometry
CD8α	100723, 100722	BioLegend	Flow cytometry
F4/80	123109	BioLegend	Flow cytometry
FOXP3	35-5773-82	eBiosciences	Flow cytometry
IL-10	12-7101-41	eBiosciences	Flow cytometry
IL-17A	506939	BioLegend	Flow cytometry
IL-17F	517006, 517003	BioLegend	Flow cytometry
RORγt	12-6981-82	eBiosciences	Flow cytometry
NGFR	345106	BioLegend	Flow cytometry
TCRβ	109225	BioLegend	Flow cytometry
Thy1.1	202508, 202521	BioLegend	Flow cytometry

Table S2. Primer sequences. Related to Figure 5G.

qPCR primers	Sequences
mCd4 F	CTTCGCAGTTTGATCGTTTTGAT
mCd4 R	CCGGACTGAAGGTCACTTTGA
mCd19_F	GGAGGCAATGTTGTGCTGC
mCd19_R	ACAATCACTAGCAAGATGCCC
mCd11b/Itgam_F	GCTCGACACCATCGCATCTA
mCd11b/Itgam_R	TGGTACTTCCTGTCTGCGTG
mCd11c/Itgax F	CTGGATAGCCTTTCTTCTGCTG
mCd11c/Itgax R	GCACACTGTGTCCGAACTCA
mCsf2 F	TCGTCTCTAACGAGTTCTCCTT
mCsf2 R	CGTAGACCCTGCTCGAATATCT
mGapdh F	AATGTGTCCGTCGTGGATCT
mGapdh R	CATCGAAGGTGGAAGAGTGG
mIfng F	ACAGCAAGGCGAAAAAGGATG
mIfng R	TGGTGGACCACTCGGATGA
mIl10 F	GCTGGACAACATACTGCTAACC
mIl10 R	ATTTCCGATAAGGCTTGGCAA
mIl12p40 F	TGCCAGGAGGATGTCACCT
mIl12p40 R	GGCGGGTCTGGTTTGATGAT
mIl17a F	TTTAACTCCCTTGGCGCAAAA
mIl17a R	CTTTCCCTCCGCATTGACAC
mIl17f F	TCCCCTGGAGGATAACACTG
mIl17f R	GGGGTCTCGAGTGATGTTGT
mIl1b F	GAAATGCCACCTTTTGACAGTG
mIl1b R	CTGGATGCTCTCATCAGGACA
mIl22 F	CCGAGGAGTCAGTGCTAAGG
mIl22 R	CATGTAGGGCTGGAACCTGT
mIl23a F	CAGCAGCTCTCTCGGAATCTC
mIl23a R	TGGATACGGGGCACATTATTTTT
mIl23r F	AGAGACACTGATTTGTGGGAAAG
mIl23r R	GTTCCAGGTGCATGTCATGTT
mIl6 F	TCTATACCACTTCACAAGTCGGA
mIl6 R	GAATTGCCATTGCACAACTCTTT
mTgfb F	CAACAATTCCTGGCGTTACC
mTgfb R	GCTGAATCGAAAGCCCTGTA
